# Galactosylated hydroxyl‐polyamidoamine dendrimer targets hepatocytes and improves therapeutic outcomes in a severe model of acetaminophen poisoning‐induced liver failure

**DOI:** 10.1002/btm2.10486

**Published:** 2023-02-08

**Authors:** Joshua E. Porterfield, Rishi Sharma, Ambar Scarlet Jimenez, Nirnath Sah, Sean McCracken, Lucia Zhang, Hyoung‐Tae An, Seulki Lee, Sujatha Kannan, Anjali Sharma, Rangaramanujam M. Kannan

**Affiliations:** ^1^ Center for Nanomedicine, Department of Ophthalmology Wilmer Eye Institute, Johns Hopkins University School of Medicine Baltimore Maryland USA; ^2^ Department of Chemical and Biomolecular Engineering Johns Hopkins University Baltimore Maryland USA; ^3^ Department of Biomedical Engineering Johns Hopkins University Baltimore Maryland USA; ^4^ Department of Radiology Johns Hopkins University School of Medicine Baltimore Maryland USA; ^5^ Department of Anesthesiology and Critical Care Medicine Johns Hopkins University School of Medicine Baltimore Maryland USA; ^6^ Hugo W. Moser Research Institute at Kennedy Krieger, Inc. Baltimore Maryland USA; ^7^ Present address: Department of Chemistry Washington State University Pullman Washington USA

**Keywords:** acetaminophen poisoning, dendrimers, drug delivery, hepatocytes targeting, liver injury, *N*‐acetyl cysteine

## Abstract

Toxicity to hepatocytes caused by various insults including drugs is a common cause of chronic liver failure requiring transplantation. Targeting therapeutics specifically to hepatocytes is often a challenge since they are relatively nonendocytosing unlike the highly phagocytic Kupffer cells in the liver. Approaches that enable targeted intracellular delivery of therapeutics to hepatocytes have significant promise in addressing liver disorders. We synthesized a galactose‐conjugated hydroxyl polyamidoamine dendrimer (D4‐Gal) that targets hepatocytes efficiently through the asialoglycoprotein receptors in healthy mice and in a mouse model of acetaminophen (APAP)‐induced liver failure. D4‐Gal localized specifically in hepatocytes and showed significantly better targeting when compared with the non‐Gal functionalized hydroxyl dendrimer. The therapeutic potential of D4‐Gal conjugated to *N‐*acetyl cysteine (NAC) was tested in a mouse model of APAP‐induced liver failure. A single intravenous dose of a conjugate of D4‐Gal and NAC (Gal‐d‐NAC) improved survival in APAP mice, decreased cellular oxidative injury and areas of necrosis in the liver, even when administered at the delayed time point of 8 h after APAP exposure. Overdose of APAP is the most common cause of acute hepatic injury and liver transplant need in the United States, and is treated with large doses of NAC administered rapidly within 8 h of overdose leading to systemic side effects and poor tolerance. NAC is not effective when treatment is delayed. Our results suggest that D4‐Gal is effective in targeting and delivering therapies to hepatocytes and Gal‐D‐NAC has the potential to salvage and treat liver injury with a broader therapeutic window.

## 1 INTRODUCTION

Acute liver failure occurs due to rapid and catastrophic injury to hepatocytes with loss of function and is accompanied by coagulopathy and encephalopathy rapidly progressing to death. Viral hepatitis and drug toxicities including acetaminophen (APAP) overdose are the most common causes of acute liver failure.^1^, ^2^ APAP overdose is responsible for almost 50% of acute liver failure cases^1^, ^2^ and 7% of liver transplants,^3^ representing an enormous burden on the healthcare system. APAP is an over‐the‐counter analgesic, but, because it is easy to access and incorporated into many medications, has been a significant source of both intentional^4^ and unintentional^5^ overdose at almost equivalent rates.^6^ This commonly used drug becomes toxic instead of salutatory when taken in large quantities as the tertiary pathway of APAP metabolism in the liver depletes endogenous glutathione levels, allowing for the accumulation of *N*‐acetyl‐*p*‐benzoquinone imine (NAPQI) in hepatocytes, which creates protein adducts and causes mitochondrial fission and eventual hepatocyte apoptosis.^7^ Fortunately, through treatment with intravenous or oral *N*‐acetyl cysteine (NAC) soon after overdose, liver failure can be prevented or reversed in most acute cases.^8^ However, even with liver transplants and NAC therapy, the mortality rate of patients with APAP‐induced acute liver failure is currently at 30%.^9^ The benefits of NAC therapy when a patient arrives in the emergency room more than 14 h after the overdose are questionable and unproven.^10^ These patients who are not candidates for NAC therapy due to severe overdose or late arrival at the hospital represent a significant underserved population. Intentional overdoses are high doses^11^ and the symptoms of APAP‐induced acute liver failure do not manifest until hours after ingestion,^2^ making timely transport to the hospital unlikely in cases of unintentional overdose. Therapies that specifically target hepatocytes may help rescue liver cell function in severe cases.

A promising branch of novel hepatic therapeutics is nanoparticle‐mediated drug delivery.^12^, ^13^, ^14^, ^15^, ^16^, ^17^ However, very few nanoparticles actually are taken up into liver cells in a meaningful way as they are rapidly cleared from circulation by Kupffer cells (liver macrophages) through the mononuclear phagocyte system and hepatocytes through hepatobiliary clearance as part of the reticuloendothelial system.^18^ The function of the reticuloendothelial system is critical for the removal of toxins and foreign bodies from the bloodstream but proves a major hindrance to the delivery of drugs to hepatocytes. Targeted nanomedicine is one way for overcoming hepatobiliary clearance by incorporating ligands to the nanoparticles' surfaces that actively bind with receptors expressed on hepatocytes, such as hepatic transferrin, G‐coupled protein, and asialoglycoprotein receptors (ASGPR).^19^, ^20^, ^21^, ^22^ ASGPR is unique in that it is only expressed on hepatocytes and has a high binding affinity for galactose, glucose, and lactose sugars; however, there are limits to the size and dosage of particle uptake by hepatocytes via ASGPR.^23^

Sugars are of particular import to dendrimer nanoparticles because the multifunctional nature of the dendrimer allows it to participate in both multivalent binding^24^, ^25^ and the glycoside cluster effect, which describes the ability of multivalent carbohydrates to bind with greater strength to their target receptors than would be predicted even after corrections for molar concentration and valence.^26^ Researchers have previously shown that a linear increase in the number of monovalent ligands on the dendrimer surface results in a nonlinear increase of receptor‐specific binding in the case of carbohydrate‐coated dendrimers.^27^, ^28^ However, some of these studies were performed on dendrimers that may be considered toxic when delivered systemically.^29^ Hydroxyl‐terminated polyamidoamine (PAMAM) dendrimers are a class of nanoparticles with very few reported systemic toxicities,^30^, ^31^, ^32^ which are further minimized above generation 3 when the end groups provide ample shielding of the ethylenediamine core. Generation 4 hydroxyl‐terminated PAMAM dendrimers (D4‐OH) specifically have been shown to perfuse freely through tissue, clear intact through the kidneys, and localize only in actively phagocytic macrophages and microglia in many animal models.^33^, ^34^, ^35^ Additionally, D4‐OH is far smaller than the fenestrations in the sinusoidal epithelium, allowing for free access to liver hepatocytes.^36^ The diffusive and biocompatible qualities of these dendrimers make them good candidates for developing targeted drug delivery strategies, and it has already been shown that the introduction of targeting ligands on these dendrimers can modify which organs, cells,^37^ and even subcellular organelles^38^, ^39^ the dendrimer localizes within upon systemic administration without impeding the dendrimers' inherent transport processes.

In this study, we have developed a generation 4 hydroxyl‐terminated PAMAM dendrimer modified to display 12 galactose molecules on the surface. We show that the surface galactose sugars create a multivalent binding effect to ASGPR, allowing the dendrimer to selectively target and internalize in hepatocytes in vitro and in vivo. This hepatocyte‐targeting dendrimer was then conjugated to NAC and efficacy was assessed in a mouse model of severe APAP poisoning with a high rate of mortality.

## 2 RESULTS AND DISCUSSION

### 2.1 Synthesis of dendrimer conjugates

**FIGURE 1:**
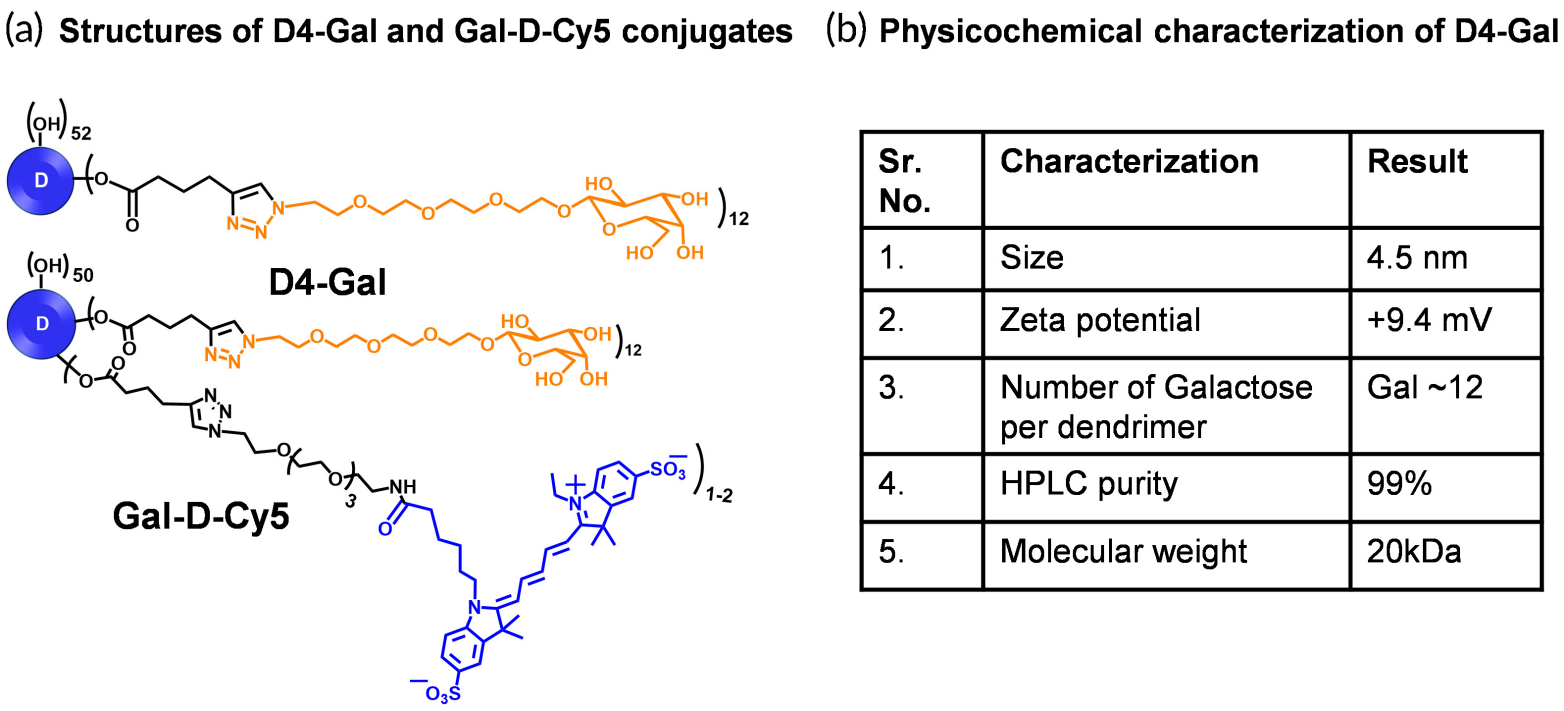
(a) The structures of dendrimer‐galactose (D4‐Gal) and fluorescently labeled Gal‐D‐Cy5; (b) Table presenting the physicochemical properties of dendrimer‐galactose conjugate.

For the investigation of the effect of galactosylation on dendrimer binding and transport kinetics, we appended galactose to the surface of D4‐OH (D4‐Gal) followed by the additional attachment of Cy5 fluorophore (Gal‐D‐Cy5) to make D4‐Gal observable in vitro and in vivo. The synthesis of both D4‐Gal and Gal‐D‐Cy5 was achieved using our recently published protocols.^40^, ^41^ The structures of both D4‐Gal and Gal‐D‐Cy5 along with their physicochemical properties are presented in Figure 1a,b. D4‐Gal contains on an average 12 molecules of β‐d‐galactose on the hydroxyl dendrimer surface with an additional attachment of 1–2 molecules of near infrared dye cyanine 5 (Cy5) for Gal‐D‐Cy5. The size of D4‐Gal is 4.5 nm and the zeta potential is nearly neutral.^41^

**SCHEME 1:**
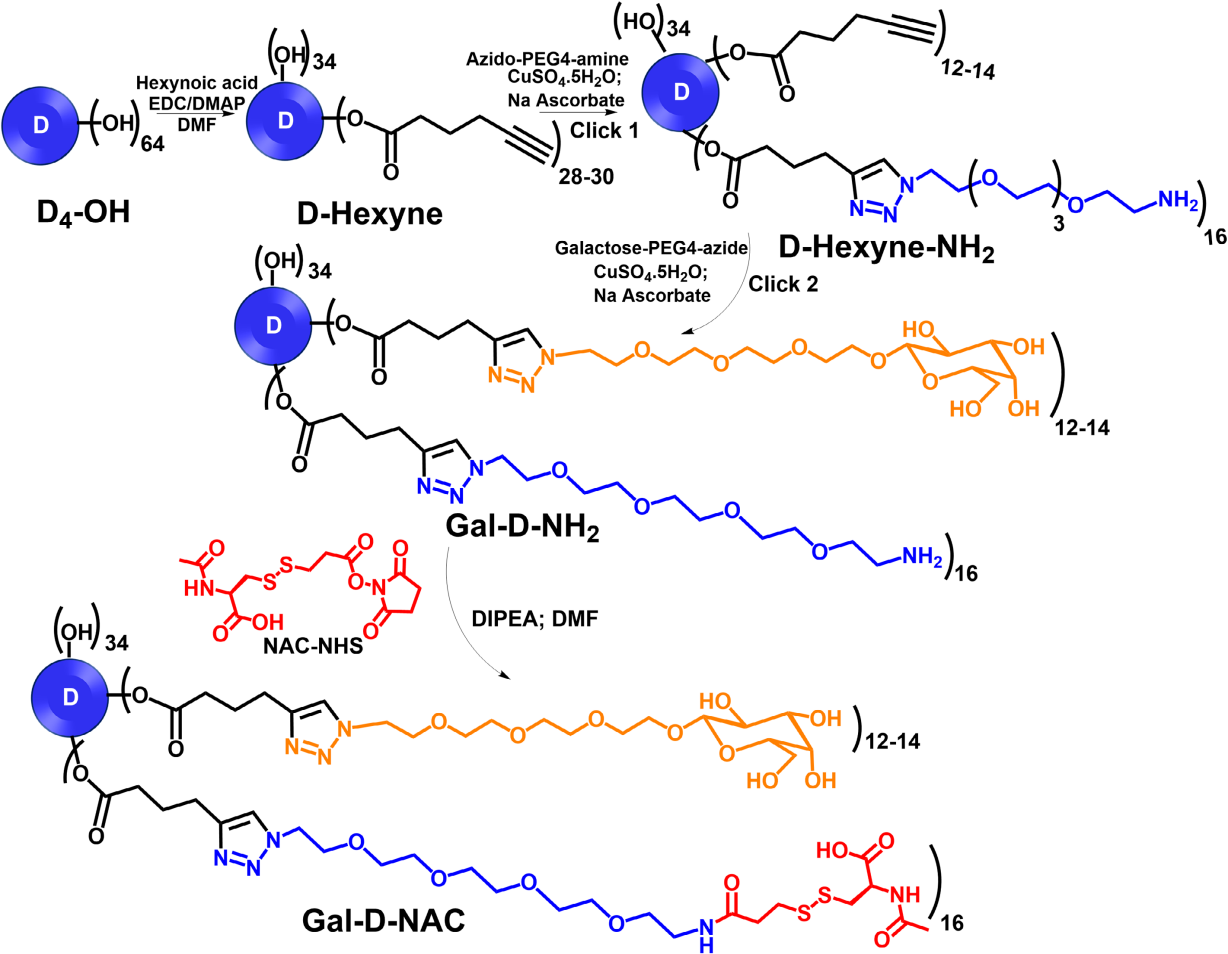
Schematic representation of step‐wise synthesis of dendrimer containing galactose and *N*‐acetyl cysteine (NAC) molecules (Gal‐D‐NAC)

**FIGURE 2:**
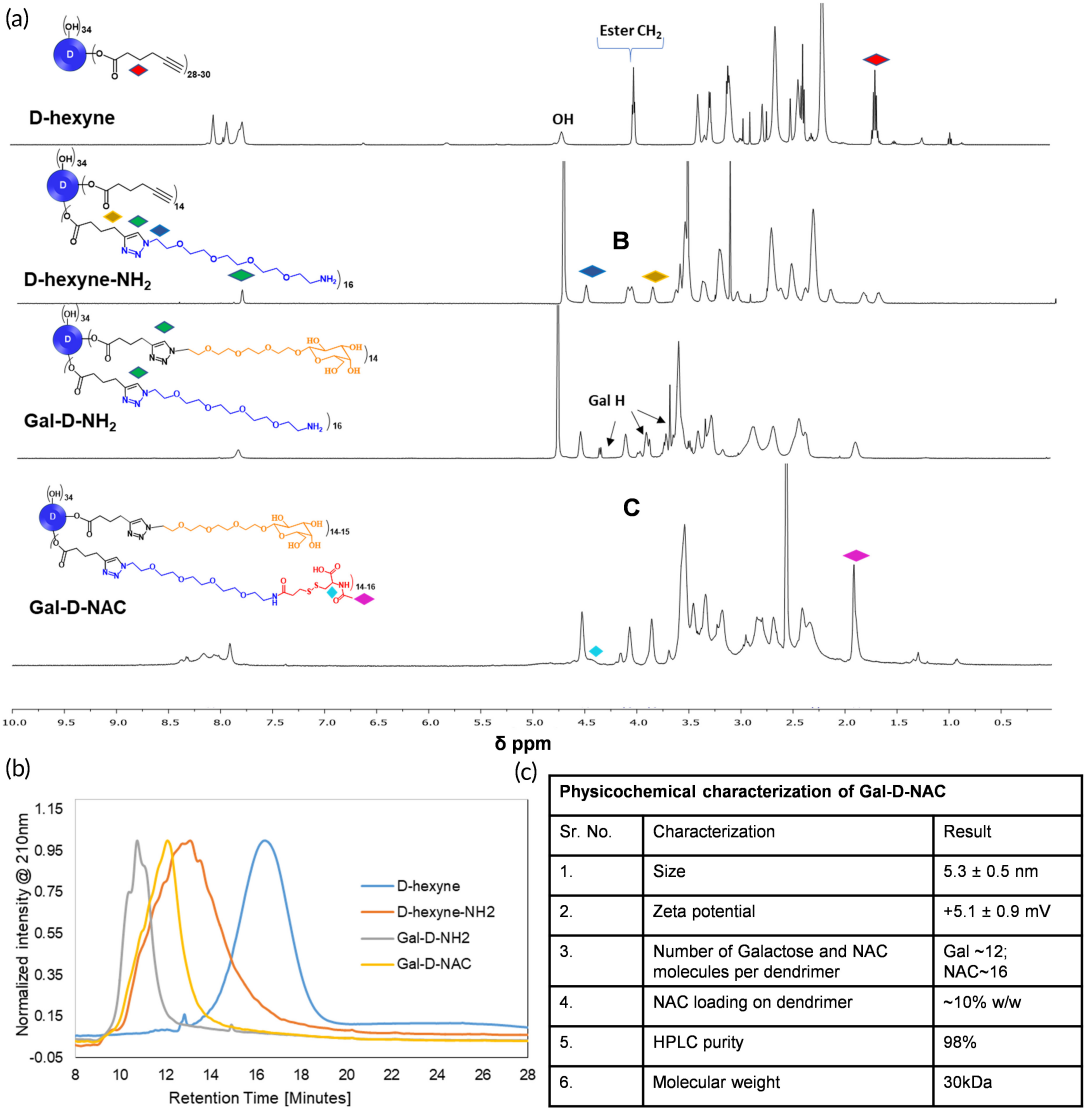
(a). ^1^H NMR of dendrimer intermediates and the final Gal‐D‐NAC conjugates depicting the presence of characteristic protons; (b) high‐performance liquid chromatography (HPLC) traces showing a shift in retention time at each synthetic step (RT‐d‐hexyne: 16.4 min; d‐hexyne‐NH_2_: 13.0 min; Gal‐D‐NH_2_: 10.7 min; Gal‐D‐NAC: 12.0 min); and (c) table representing the physicochemical properties of Gal‐D‐NAC

Further, to evaluate the therapeutic potential of D4‐Gal for the delivery of a potent antioxidant and anti‐inflammatory drug NAC to hepatocytes for the treatment of APAP poisoning, we synthesized a NAC‐loaded D4‐Gal conjugate, Gal‐D‐NAC (Scheme 1) with an average of ~12 molecules of galactose on the surface and ~16 molecules of NAC connected to the dendrimer via a cleavable, glutathione‐sensitive disulfide linker. The synthesis of Gal‐D‐NAC was achieved using highly efficient copper(I) catalyzed alkyne‐azide click (CuAAC) reaction. D4‐OH was modified with hexynoic acid through *N*‐(3‐dimethylaminopropyl)‐*N*′‐ethylcarbodiimide hydrochloride (EDC)/4‐(dimethylamino)pyridine (DMAP) coupling esterification to create d‐hexyne with ~30 alkyne arms. The integration comparison of ester —CH_2_ protons at 4.01 ppm to dendrimer internal amide protons at 8.5–7.5 ppm was used to calculate the number of alkyne arms (Figure 2a). An azido‐PEG‐4‐amine linker for eventual NAC conjugation was then attached to ~15–16 of the hexyne groups through a CuAAC reaction, resulting in d‐hexyne‐NH_2_. The appearance of triazole H along with a shift in methylene *H* next to triazole ring confirms the click reaction (Figure 2a). The remaining hexyne groups were then reacted with β‐galactose‐TEG‐azide (Scheme 1) to create Gal‐D‐NH_2_, which was further reacted with NAC‐NHS ester through an activated acid‐amine coupling reaction to yield the final product, Gal‐D‐NAC having ~12–14 galactose and 15–16 NAC on the surface. The ^1^H NMR clearly showed the presence of galactose and NAC protons. The purity of the final products and intermediates was characterized with high performance liquid chromatography (HPLC) (Figure 2b and Figures S3, S5, and S7). The HPLC chromatogram showed a shift in the retention time at each step and the final Gal‐D‐NAC conjugate demonstrated a purity of 98%. The physicochemical properties of Gal‐D‐NAC are presented in Figure 2c. The size and zeta potential of Gal‐D‐NAC (size: 5.3 nm; zeta potential: +5 mV; Figure 2c and Figures S9 and S10) did not diverge greatly from those of D4‐Gal (size: 4.5 nm, zeta potential: +9 mV; Figure 1b) indicating that the addition of NAC will likely not have a major effect on the biodistribution of D4‐Gal, as it has been previously shown that D‐Cy5 and NAC‐D‐Cy5 have similar localization in vivo.^34^

### 2.2 Defining D4‐Gal binding affinity and hepatocyte uptake in vitro

**FIGURE 3:**
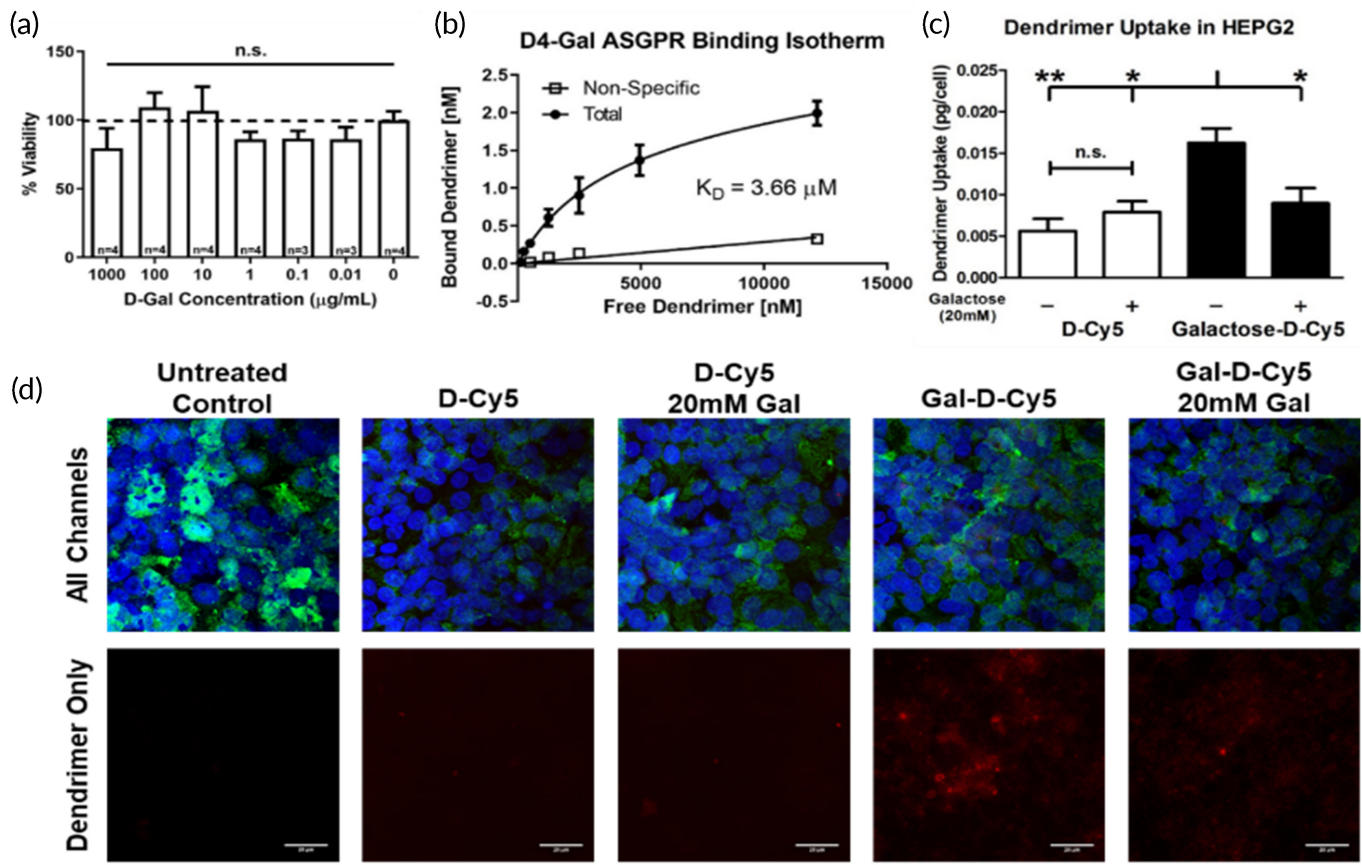
Assessment of D4‐Gal ASGPR binding and hepatocellular uptake in vitro. (a) MTT assay showing lack of toxicity of the D4‐Gal compound at concentrations up to 1000 μg/ml in HEPG2 cells. (b) Direct evaluation of binding affinity (*K*_*D*_) of D4‐Gal to ASGPR through incubation with cellularly expressed receptors in vitro. *K*_*D*_ was determined to have a value of 3.7 μM. (**c)** Dendrimer uptake in HEPG2 culture shows that Gal‐D‐Cy5 has an increased ability to internalize in HEPG2 cells, which is returned to the baseline uptake of unmodified dendrimer upon co‐incubation with excess free galactose sugar at 20 mM concentrations. **(d)** Confocal imaging of HEPG2 cells shows increased internalization of Gal‐D‐Cy5 than is reduced to D‐Cy5 levels with free galactose co‐incubation. ASGPR (green), dendrimer (red), Hoechst 33342 (blue). Representative images shown. Scale bars are 25 μm

The primary design criterion for D4‐Gal was multivalent binding to ASGPR for internalization in hepatocytes, so we first determined the dendrimer's binding affinity for ASGPR, after assuring that it was nontoxic up to 1000 μg/ml as determined by the MTT (3‐(4,5‐di**m**ethyl**t**hiazol‐2‐yl)‐2,5‐diphenyl**t**etrazolium bromide) dye cell viability assay (Figure 3a). A D4‐OH‐NAC compound (OP‐101) is currently undergoing clinical trials,^42^ and the compound remains nontoxic at therapeutic levels (NCT04321980, NCT03500627, and NCT04458298). We believe that the addition of nontoxic sugar to the dendrimer will not change this result in hepatocytes, but the effects of long‐term dendrimer accumulation in hepatocytes will require further study prior to translation.

To quantify the binding affinity of D4‐Gal for ASGPR, we utilized a cell surface binding assay^40^, ^43^ on HEPG2 cells, which express on the order of 100,000 ASGPR per cell.^44^ This method was chosen as it has previously been reported that binding affinity to ASGPR can change over 100‐fold between free receptor systems, like surface plasmon resonance, and cell‐based assays.^45^ Based on the total binding isotherm and the correlated nonspecific binding quantification through the introduction of ASGPR blocking peptide, we determined the binding affinity, *K*_*D*_, of D4‐Gal to ASGPR to be 3.66 μM (Figure 3b, *r* ^2^ = 0.9192 total, *r* ^2^ = 0.8297 nonspecific, 95% CI: 2.025 < *K*_*D*_ <6.562 μM, *n* = 3) as opposed to D4‐OH, which indicated no preferential binding to ASGPR. *K*_*D*_ of free galactose is estimated to be between 2 and 5 mM,^46^ making the binding of D4‐Gal to ASGPR about 1000‐fold stronger than free galactose sugar. We attribute the observed improvement in binding affinity to the impact of both multivalency and the cluster glycoside effect.^24^, ^25^

Introducing the capacity to bind to ASGPR to the dendrimer was encouraging, so we next investigated the impact of galactosylation on dendrimer uptake into HEPG2 cells in culture. Following 24 h of treatment with medium containing either Gal‐D‐Cy5 or D‐Cy5, HEPG2 cells were found to have a significantly increased uptake of Gal‐D‐Cy5 at 0.016 pg dendrimer/cell as opposed to unmodified D‐Cy5, which was only present at levels of 0.006 pg/cell (*p* = 0.007; Figure 3c, two‐way ANOVA for dendrimer and treatment *F*(1,12) = 10.43, *p* = 0.0072, *n* = 4). We then pretreated and co‐incubated HEPG2 cells with free galactose sugar to determine whether increased uptake was in fact due to enhanced binding to ASGPR. Free galactose pretreatment reduced Gal‐D‐Cy5 uptake to 0.0097 pg/cell, significantly lower than untreated Gal‐D‐Cy5 uptake (by ~40%, *p* = 0.0223), while having no impact on non‐Gal‐functionalized D‐Cy5 uptake. Based on the results of the binding study, the presence of 20,000‐fold more free ligand than that present on the dendrimer would be sufficient to quench ASGPR‐mediated uptake of Gal‐D‐Cy5, which was observed as co‐incubation with galactose resulted in uptake of Gal‐D‐Cy5 equivalent to that of D‐Cy5. Appreciable, qualitatively similar uptake of free galactose‐inhibited Gal‐D‐Cy5 and D‐Cy5 indicates that the modified dendrimer still maintains its ability to enter cells through nonspecific fluid phase endocytosis,^47^ which has interesting implications on the development of future targeted and drug‐loaded D4‐OH dendrimer conjugates. Previous studies with galactose dendrimers have shown that both co‐incubation with galactose and treatment with chlorpromazine, which blocks clathrin‐mediated endocytosis, as is the case with ASGPR, diminish dendrimer uptake by similar amounts,^40^ giving additional credence to the assertion that the increased HEPG2 uptake observed here is due to ASGPR binding and internalization. The dendrimer uptake was additionally observed through confocal microscopy (Figure 3d), where it was evident that Gal‐D‐Cy5 signal was much more prevalent than when co‐treated with galactose and D‐Cy5 in any condition. Gal‐D‐Cy5 signal is also imaged throughout the cytoplasm, an indicator that the dendrimer escapes from endosomes once internalized in the cell, possibly via the proton sponge effect,^48^ as nanoparticles entrapped in vesicles would appear punctate in images.

### 2.3 Pharmacokinetics of intravenous Gal‐D‐Cy5 in vivo in healthy mice

**FIGURE 4:**
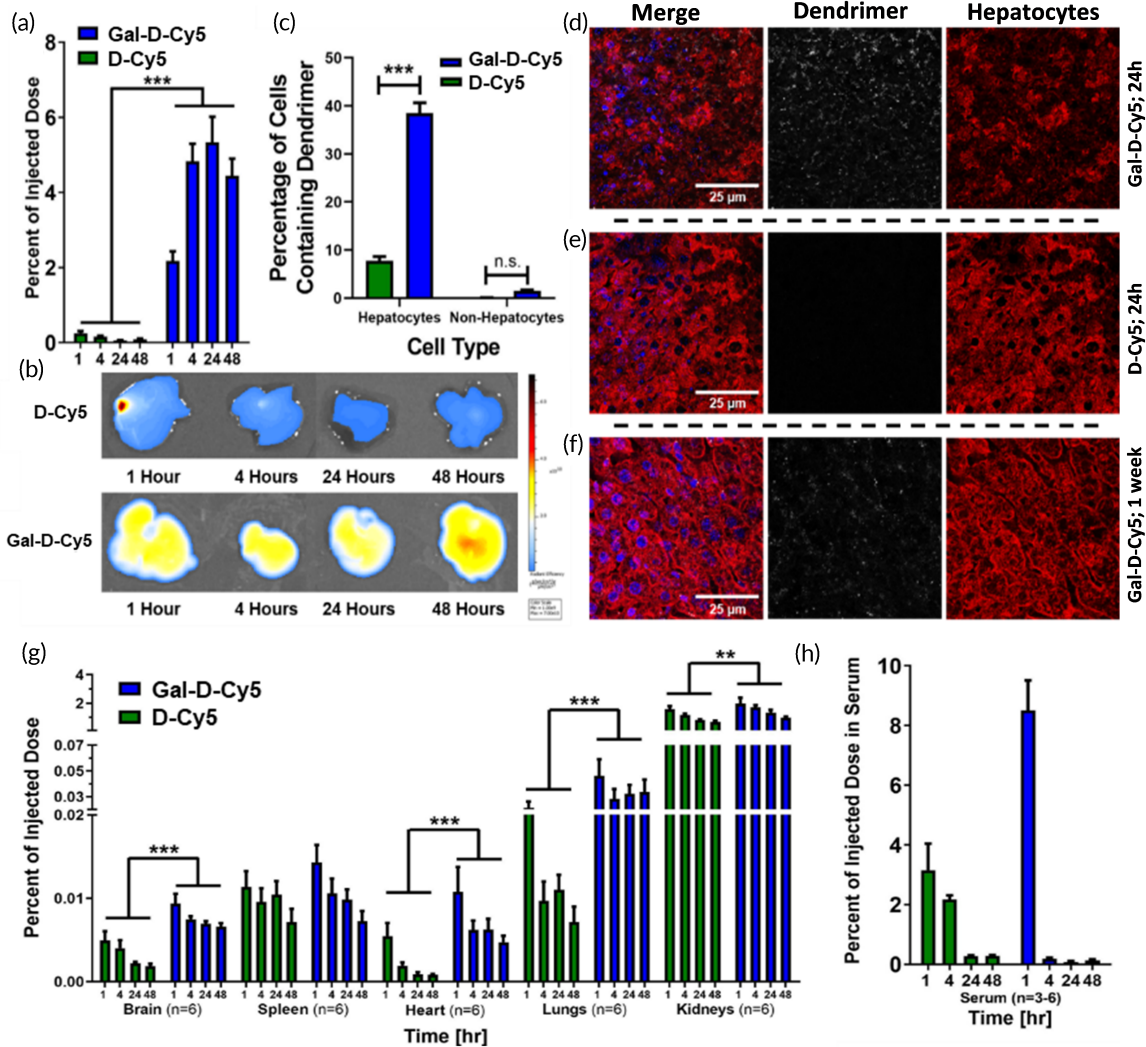
Liver and hepatocyte localization of Gal‐D‐Cy5 upon systemic administration. (a) Gal‐D‐Cy5 administered to healthy C57BL6 mice via tail vein injection localizes significantly more in the liver as opposed to unmodified D‐Cy5, which clears quickly from hepatic tissue (*n* = 4–6). (b) IVIS® imaging of ex vivo mouse livers shows dramatically increased Cy5 fluorescence signal from dendrimer localization in the Gal‐D‐Cy5 injected tissue over the D‐Cy5 injected tissue. (c) Flow cytometry of primary mouse livers shows that Gal‐D‐Cy5 uptake is highly specific to hepatocytes unlike D‐Cy5 (*n* = 3). (d) Gal‐D‐Cy5 localization in mouse liver 24 h after systemic administration via tail vein. Gal‐D‐Cy5 (white) localizes specifically inside the border of hepatocytes (serum albumin, red) and not in the unstained sinusoidal spaces as is typical of other nanoparticles. (e) D‐Cy5 localization in mouse liver 24 h after systemic administration via tail vein. D‐Cy5 (white) has such low localization in liver that it was not readily visible through confocal microscopy. (f) Gal‐D‐Cy5 (white) shows persistent, robust localization in liver hepatocytes (red) 1 week after systemic administration. Nuclei are stained with Hoechst 33342 (blue). Scale bars are 25 μm. (g) Gal‐D‐Cy5 full body pharmacokinetics. Gal‐D‐Cy5 clears from nonhepatic tissue at rates comparable to that of unmodified D‐Cy5 with no tissue other than kidneys demonstrating uptake of greater than 0.1% of the initial injected dose 48 h after administration. There is statistically significantly greater uptake of Gal‐D‐Cy5 over D‐Cy5 in the healthy brain, heart, lungs, and kidneys, which may be due to sugar receptors in these organs, but the difference was not enough to observe differences in tissue imaging (*n* = 6 for all treatments and times). (h) Gal‐D‐Cy5 also clears more rapidly from serum, potentially due to its high accumulation in liver tissue (*n* = 6 for all treatments and times)

Based on these promising in vitro results, we investigated the in vivo hepatocellular targeting of D4‐Gal conjugate and compared it to D4‐OH, which is known to minimally distribute to the liver and is cleared rapidly through the kidneys due to its size^49^ (diameter of ~4 nm for D4‐OH that increases slightly to ~4.5 nm with the conjugation of galactose to the surface). Healthy C57BL6 mice were administered 55 mg/kg of D‐Cy5 or Gal‐D‐Cy5 via tail vein injection with biodistribution assessed at 1, 4, 24, and 48 h. The choice of healthy C57BL6 mice was to aid in future studies on APAP poisoning as the C57BL6 mouse is the superior model choice due to their similar metabolism of APAP by cytochrome p450 as in humans,^50^ while observing interaction of the dendrimer with healthy liver tissue. Mouse liver tissue was homogenized in methanol to extract the dendrimer, which revealed a significant presence of Gal‐D‐Cy5 in the liver. Gal‐D‐Cy5 uptake in liver was significantly greater than that of D‐Cy5 at all times (Figure 4a, two‐way ANOVA, *F*(1,34) = 274.8, *p* < 0.0001, *n* = 4–6), with a ~90‐fold difference between Gal‐D‐Cy5 and D‐Cy5 at the 24 h, where peak uptake was seen. Localization of Gal‐D‐Cy5 in the liver is maintained between 24 and 48 h at ~5% of the total injected dose (24 vs. 48 h Gal‐D‐Cy5, Tukey's multiple comparison *t*‐test, *p* = 0.6864), indicating sustained delivery to the liver unlike most liver‐targeting nanoparticles that typically clear more rapidly.^51^, ^52^ Fluorescent imaging of ex vivo livers (Figure 4b) using the IVIS® Spectrum optical imaging device enabled visualization of the difference between D‐Cy5 and Gal‐D‐Cy5 accumulation and confirmed that Gal‐D‐Cy5 localized homogeneously throughout the liver.

Future therapies for liver disorders, such as drug‐induced liver failure, hepatocellular carcinoma, and hepatitis, will benefit from drug localization in hepatocytes, or have hepatocyte‐specific mechanisms of action,^46^ so we further analyzed the cellular localization of Gal‐D‐Cy5 within the liver through both flow cytometry (Figure 4c) and confocal imaging (Figure 4d–f). Flow cytometry of liver cells isolated from mice injected with either D‐Cy5 or Gal‐D‐Cy5 was labeled for hepatocytes with ASGPR antibody (Figure 4c). We observed that ~40% of all live hepatocytes were positive for Gal‐D‐Cy5 24 h after injection. This uptake is significantly greater than Gal‐D‐Cy5 in cells that were not hepatocytes which showed less than 5% uptake (two‐way ANOVA *F*(1,12) = 604.5, *p* < 0.0001, *n* = 3, Tukey's multiple comparison *t*‐test *p* < 0.0001), or D‐Cy5 in either hepatocytes or nonhepatocytes (*p* < 0.0001 in both cases). Confocal imaging of Gal‐D‐Cy5 in the liver tissue sections stained to identify both hepatocytes and sinusoidal endothelial cells (SECs) showed that Gal‐D‐Cy5 signal almost exclusively colocalized with hepatocytes. This is in stark contrast to D‐Cy5, which was not present in appreciable quantities in the liver. Other nanoparticles that accumulate in the liver are reported to only localize in the sinusoid endothelial cells, as they are removed from systemic circulation through the mononuclear phagocyte system.^37^, ^53^, ^54^ Interestingly, Gal‐D‐Cy5 signal was still easily observed in hepatocytes 1 week after injection (Figure 4f), which is uniquely favorable for the sustained delivery of drugs. These data conclusively show that systemically injected D4‐Gal specifically targets hepatocytes and is retained intracellularly for at least up to 1 week following injection.

To demonstrate liver specificity and determine whether there is significant uptake or retention in off‐target (nonhepatic) tissue, we further analyzed the pharmacokinetics of Gal‐D‐Cy5 and D‐Cy5 in plasma and other organs in mice (Figure 4g,h). Two‐way ANOVA analysis indicated that Gal‐D‐Cy5 was present in somewhat higher quantities than D‐Cy5 in the brain (still both compounds showed <0.01% of the injected dose in brain) (*F*(1,40) = 73.57, *p* < 0.0001), heart (*F*(1,38) = 28.47, *p* < 0.0001), lungs (*F*(1,40) = 20.53, *p* < 0.0001), kidneys (*F*(1,40) = 10.48, *p* = 0.0024), and plasma (*F*(1,24) = 3.994, *p* = 0.0571); however, in none of these organs was there a main effect of the interaction between dendrimer and time (*p* = 0.4580, 0.9093, 0.9433, and 0.9516, respectively) indicating that while there was a minor increased uptake, the accumulation and clearance rates of Gal‐D‐Cy5 from these organs are not significantly different from that of D‐Cy5. The somewhat increased uptake levels may be due to sugar receptors, such as sodium‐glucose cotransporter type 1 and glucose transporter 1, that are expressed throughout the body and have slight affinities for galactose,^55^, ^56^ but any increase in uptake was minor, with no other organ, but the kidneys containing more than 0.05% of the total injected dose at 48 h, and the kidneys containing less than 1% ID, which is no different than D‐Cy5 (Tukey's multiple comparison *t*‐test, *p* = 0.9420). There was a main effect of interaction in plasma (*F*(3,24) = 18.57, *p* < 0.0001), which was most likely due to the reduction of Gal‐D‐Cy5 concentration in plasma. This may have arisen from the rapid accumulation of Gal‐D‐Cy5 in the liver, as opposed to D‐Cy5 that is gradually filtered out by the kidney proximal tubules over 24 h.

**FIGURE 5:**
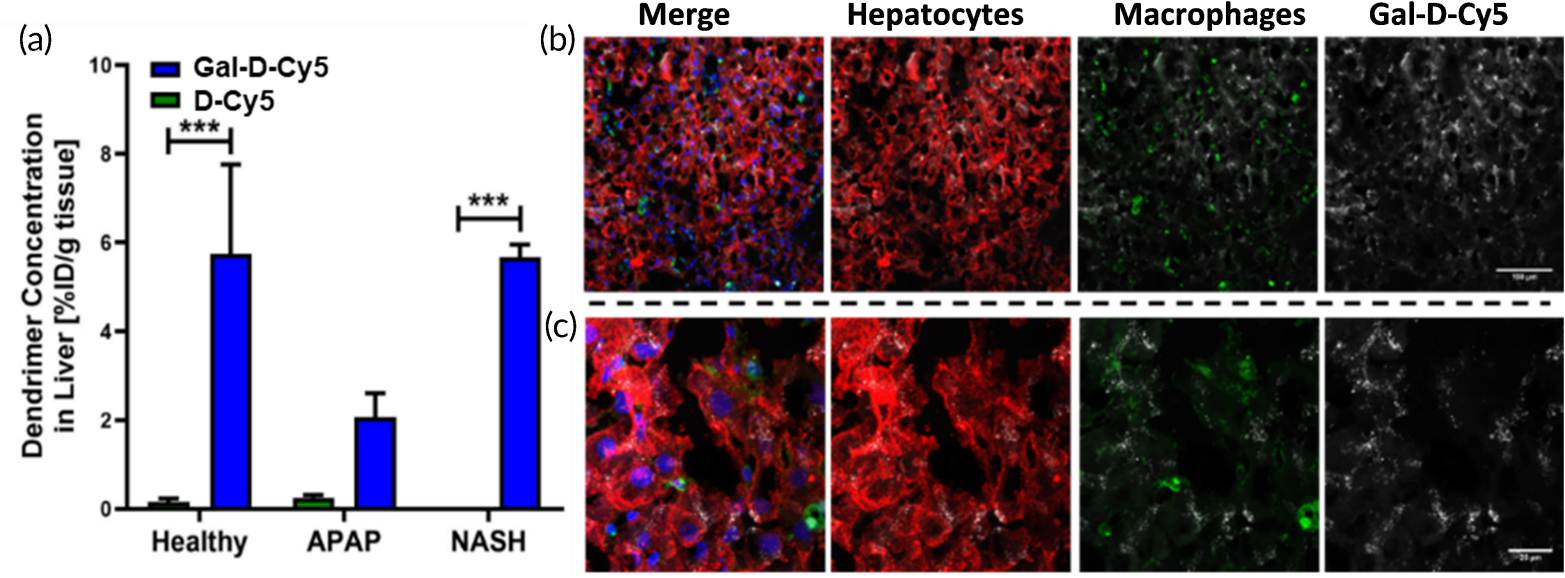
Uptake of fluorescent dendrimers in animal disease models. (a) Gal‐D‐Cy5 (*n* = 6, 3, 2) and D‐Cy5 (*n* = 6, 2, 2) uptake in healthy mice, mice 24 h following an overdose of acetaminophen (APAP), and rats fed a high‐fat methionine and choline‐deficient diet for 6 weeks to induce nonalcoholic steatohepatitis (NASH). (b) Gal‐D‐Cy5 (white) localization in a rat model of NASH is perfuse throughout the liver tissue, not specific to liver macrophages (lectin, green). (c) High magnification of the same livers shows that Gal‐D‐Cy5 (white) is concentrated in hepatocytes (ASGR, red). Nuclei are stained with Hoechst 33342 (blue).

The high specificity of Gal‐D‐Cy5 to liver hepatocytes, with relatively rapid clearance from the rest of the body makes it a desirable candidate for drug delivery to hepatocytes. We further wanted to investigate the ability of Gal‐D‐Cy5 to maintain this liver‐targeting capability in the context of liver disease, so we determined liver uptake of both Gal‐D‐Cy5 and D‐Cy5 in two different models of liver disease: a rat model of high‐fat methionine choline‐deficient (HF‐MCD) diet‐induced nonalcoholic steatohepatitis (NASH) and a mouse model of APAP‐induced liver failure. Irrespective of the species and the type of liver injury, we found that Gal‐D‐Cy5 continued to outperform D‐Cy5 with 5.66 %ID/g tissue localizing in the NASH liver and 2.06 %ID/g tissue in the APAP overdose model, which is compared to <0.3 %ID/g tissue of D‐Cy5 being taken up by the liver in either model (Figure 5a). There was a main effect of disease and dendrimer (two‐way ANOVA, *F*(2,15) = 4.717, *p* = 0.0257), and the uptake of Gal‐D‐Cy5 was significantly greater than that of D‐Cy5 in the NASH model (Sidak's multiple comparison *t*‐test, *p* = 0.0007, *n* = 2), which is clearly seen in confocal imaging of rat liver sections (Figure 5b). When images were analyzed under high magnification (Figure 5c), it was also apparent that the Gal‐D‐Cy5 had maintained the same hepatocyte‐specific localization as observed in the healthy mouse tissue previously. Gal‐D‐Cy5 uptake in the APAP overdose model was reduced ~3‐fold from healthy tissue as opposed to the NASH model, which had almost equivalent uptake as compared to healthy mice. This discrepancy is most likely due to the fact that the NASH model involves highly functioning live hepatocytes as the disease has not yet progressed to fibrosis and cirrhosis, whereas just 24 h after APAP administration there is rampant tissue necrosis and hepatocyte death in the APAP overdose model,^57^ reducing the number of live hepatocytes for Gal‐D‐Cy5 to target. Due to the success in hepatocyte‐targeting in both disease models, we chose to pursue treatment in the APAP poisoning model as a proof of concept that efficacy in a model with rampant hepatic necrosis could serve as a strong basis for future studies and clinical translation.

### 2.4 Systemic Gal‐NAC conjugate (Gal‐D‐NAC) treatment improved long‐term survival in a mouse model of severe APAP poisoning

To evaluate the in vivo efficacy of our dendrimer therapy, we conjugated NAC to the surface of D4‐Gal (Gal‐D‐NAC) through a glutathione‐sensitive disulfide linker that would release once internalized in the hepatocytes but remain stable in solution and in plasma.^58^ Systemic NAC therapy is already the standard‐of‐care for the clinical presentation of APAP poisoning but becomes ineffective at later time points or with increasingly large doses of APAP as is shown by the treatment nomogram.^10^ We hypothesized that if NAC could be delivered more rapidly and directly to the hepatocytes that need it, the treatment window for severe APAP poisoning could increase, reducing mortality and the need for liver transplants.

**FIGURE 6:**
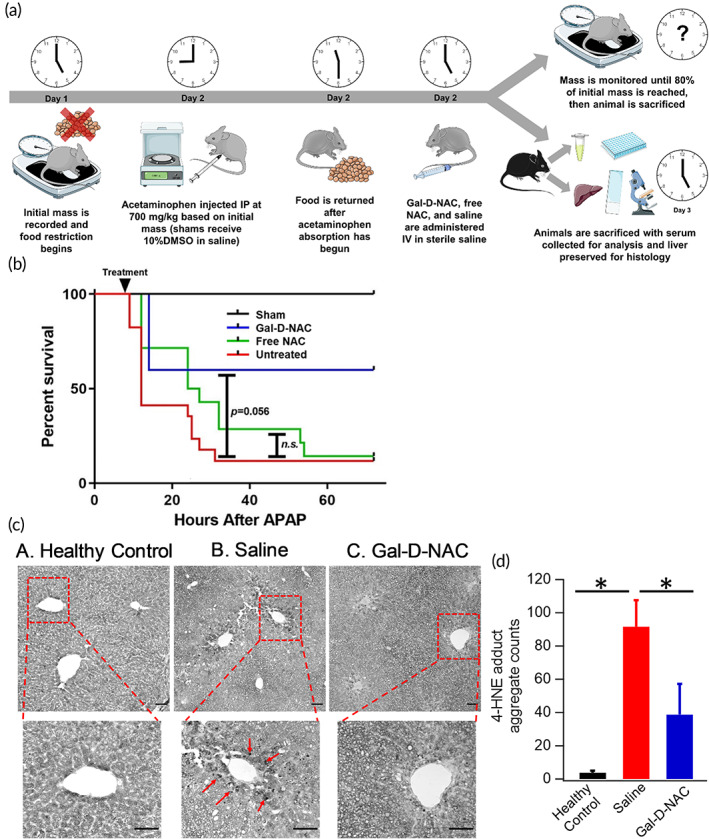
Establishment of severe acetaminophen poisoning mouse model. (a) Diagram explaining experimental set up and disease pathogenesis. APAP was injected IP at 700 mg/kg in 10% DMSO in sterile saline at a concentration of 25 mg/ml and GAL‐D‐NAC and free NAC were administered IV in sterile saline. (b) Survival curve of sham (black, *n* = 10), saline‐treated (red, *n* = 17), free NAC‐treated (green, *n* = 14), and Gal‐D‐NAC‐treated (blue, *n* = 5) animals. Over 90% of animals given acetaminophen overdose die within 96 h without treatment. (c) Mouse liver levels of 4HNE‐positive granulation in liver morphology and histology from study of Gal‐D‐NAC treatment from healthy control (c–a), untreated (c–b) and Gal‐D‐NAC‐treated animal. Arrow marks indicate the black HNE‐positive granules. Liver sections of healthy mice (c,A) do not show HNE‐positive granulation (black depositions) and mice given an overdose of acetaminophen treated with saline (c,B) showed abundant levels of HNE‐positive cellular granulation and Gal‐D‐NAC‐treated group (c,C) showed marked decrease in HNE‐positive granulation. Scale bars are 50 μm. (d) 4‐HNE granule count showed significant increase in saline treated animal compared to healthy control (*p* < 0.001). Gal‐D‐NAC treatment significantly decreased the 4‐HNE granule count indicative of decreased oxidative stress (*p* = 0.024)

First, we established a model of severe APAP poisoning through the administration of 700 mg/kg APAP i.p. to C57BL6 mice (Figure 6a). There was a high mortality rate with >90% of mice given APAP overdose dying within 72 h regardless of treatment with free NAC (Figure 6b). At this dose of APAP, both males and females demonstrated hepatotoxicity. This model therefore aims to replicate the effects of APAP overdoses that results in fulminant liver failure and are considered untreatable with the current clinical NAC regiment, when treated early. To mimic delayed treatment due to late arrival at the hospital, we also treated animals 8 h after APAP overdose as opposed to the pre‐ and co‐treatments with nanoparticles that are typically reported in this model.^59^, ^60^ Both dendrimer and free drug‐treated animals received 100 mg/kg of NAC, which was chosen because the dose is far lower than treatments shown to be effective in more mild versions of this model, and the dendrimer‐conjugate could remain soluble in a small enough volume for a single bolus dose at this concentration. Additionally, through estimates of total cells in the live and homogenous distribution, this dose of dendrimer per cell would be nontoxic according to the results earlier in this study (Figure 3a). A single treatment with Gal‐D‐NAC significantly increased survival, while free NAC did not show any improvement in survival (Figure 6b). When treated 8 h after APAP, more than 60% of Gal‐D‐NAC treated animals survived past 96 h, whereas less than 10% of free NAC and untreated animals survived. Since excess bleeding and severe coagulopathy due to the short half‐life of liver derived clotting factors can be an early mechanism of death,^61^ it is possible that some preservation of liver function after Gal‐D‐NAC treatment may have been adequate to prevent severe and fatal coagulopathy seen with acute liver failure.

We also evaluated serum levels of alanine aminotransferase (ALT) and aspartate aminotransferase (AST), enzymes in hepatocytes that catalyze the reaction from either alanine or aspartate to glutamate or pyruvate, and ALT is highly liver‐specific while AST is expressed throughout the body.^62^ These enzymes are released into the bloodstream soon after APAP overdose due to the breakdown of hepatocytes, typically peaking soon after injury as hepatocytes are actively dying and come down over time.^63^, ^64^ We analyzed serum aminotransferase values in mice sacrificed at a uniform time 24 h after treatment (32 h after APAP overdose) to see whether there were early effects of Gal‐D‐NAC therapy as would be expected of free NAC in an acute model.^65^ There was no significant difference in ALT between the groups (one‐way ANOVA, *F*(3,8) = 3.445, *p* = 0.0718) (Figure S1A). At this time point, a significant increase in AST in the untreated APAP mice was not seen when compared with healthy mice. No significant differences were seen between the groups (Figure S1B), (one‐way ANOVA, *F*(3,8) = 1.348, *p* = 0.3259). This is in accordance with previously published data where peak AST and ALT levels were observed by 16 h post‐APAP and levels started decreasing after that in a similar mouse model of APAP‐induced liver injury.^62^ It is also possible that the severely injured animals in the saline‐treated group died and the surviving animals are less injured, which may explain why the liver enzymes were not significantly different between the groups.

Mitochondrial oxidative stress and dysfunction has been described as one of the mechanisms of APAP‐induced liver injury. This can lead to DNA fragmentation, necrotic cell death and may lead to ongoing inflammation thereby perpetuating and amplifying oxidative injury. Although lipid peroxidation may not be a primary cause of cell death in APAP‐induced injury, the presence of markers of lipid peroxidation is an indicator of oxidative damage.^66^ We stained liver sections for 4 hydroxynonenal (4‐HNE) as a marker of lipid peroxidation (Figure 6c,d). 4‐HNE positive granules were only minimally detectable in liver samples from the healthy control group but were highly detectable in liver samples from untreated APAP mice, indicating increased lipid peroxidation and oxidative injury. Gal‐D‐NAC‐treated group, however, showed a reduction in 4‐HNE positive granules in the liver (Figure 6c). 4‐HNE granule count was significantly more in saline treated mouse compared to healthy control (*p* < 0.001). The Gal‐D‐NAC treatment significantly decreased the 4‐HNE granule count suggesting decreased oxidative injury (*p* = 0.024) (Figure 6d).

**FIGURE 7:**
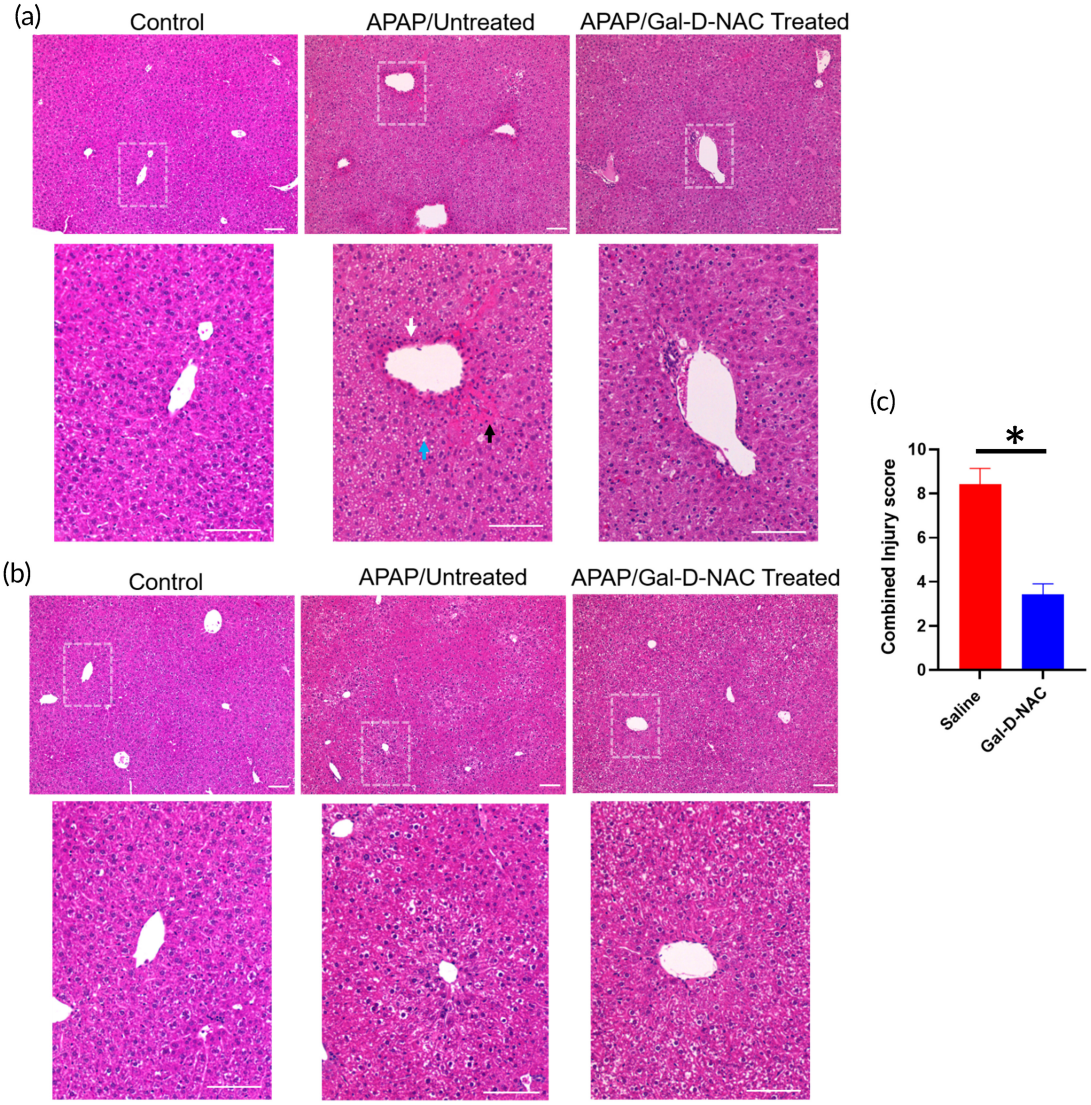
Hematoxylin and eosin staining of liver sections at 48 and 96 h after APAP exposure. (a) Sections from healthy control, APAP exposed untreated and APAP exposed Gal‐D‐NAC‐treated mice at 48 h post exposure. Arrows indicates nuclear pyknosis (white arrow), cellular vacuolation (black), and cytoplasmic eosinophilia (blue) (b) Sections from healthy control, APAP exposed untreated and APAP exposed Gal‐D‐NAC‐treated mice at 96 h post exposure. (c) Semi quantitative scoring of extent of cell death (pyknosis), hypereosinophilia, and vacuolation was graded and combined score is depicted in graph. Gal‐D‐NAC‐treated animals had lower areas of cell death/necrosis, decreased vacuolation and decreased areas of hypereosinophilia indicating improvement in degree of injury with treatment (*p < 0.05). Healthy animal did not show these features. Scale 100 μm. Detailed scoring is provided in the supplement.

We evaluated the extent of injury based on histology. Liver harvested at 48 and 96 h post‐APAP exposure was processed, sectioned, and stained with H&E. A modified Suzuki scoring was used to assess the injury and necrosis.^67^, ^68^ Liver sections from untreated mice demonstrated large areas of cellular vacuolation, cell death indicated by pyknotic nuclei, loss of cellular structure, and hypereosinophilia, which was not seen in the healthy mice and was significantly decreased in mice treated with Gal‐D‐NAC (Figure 7a–c).

Taken together these data indicate that D4‐Gal is a powerful new tool for accessing and treating hepatocytes in a targeted manner. D4‐Gal specifically targets hepatocytes very effectively and is retained in the cells, which may enable it to release the drug in a sustained manner. While specific mechanisms of action will need to be studied in the future, Gal‐D‐NAC clearly has a significant impact on mortality in severe APAP poisoning along with improvement in liver injury seen on histology. This is an early proof‐of‐concept study to demonstrate that Gal‐D‐NAC can be reliably synthesized and that it specifically targets hepatocytes delivering the drug at the site of action and producing efficacy in a model of APAP‐induced liver injury. Since similar localization is seen irrespective of the mechanism of injury, D4‐Gal can potentially be used to deliver other drugs or biologics to injured hepatocytes in other liver disorders.

## 3 MATERIALS AND METHODS

### 3.1 Dendrimer synthesis and characterization

Generation 4 hydroxyl‐terminated polyamidoamine dendrimers were purchased from Dendritech (Midland, MI) and modified through in house chemical reactions described in the results and discussion. Equipment used through reactions, purification, and validation were: 2032 Dry Fast Ultra high vacuum pump (Welch, Mt. Prospect, IL), RE200 rotary evaporator (Yamato Scientific, Tokyo, Japan), CombiFlashRf+ flash chromatography unit (Teledyne ISCO, Lincoln, NE), Nanoseries Nano‐ZS Zetasizer (Malvern, Worcester, UK), 500 MHz nuclear magnetic resonance (NMR) spectrometer (Bruker, Billerica, MA), and a HPLC assembly including 1525 Binary HPLC Pump (Waters, Milford, MA), 717 Plus Autosampler (Waters), 2475 Multi λ Detector (Waters), and In‐Line Degasser (Waters), running Empower Pro 3 software (Waters). A Symmetry C18 reverse phase column (Tosoh, Japan) having 5 μm particle size, 25 cm length, and 4.6 mm internal diameter was used for HPLC analysis. Solvents utilized for reactions, dialysis, and HPLC included methanol (VWR, Radnor, PA), acetonitrile (VWR), water (VWR), and anhydrous *N*,*N*‐dimethylformamide (DMF, Sigma‐Aldrich, St. Louis, MO). ^1^H‐NMR samples were dissolved in dimethyl sulfoxide‐d6, deuterium oxide, methanol‐d4, or chloroform‐d (Cambridge Isotope Laboratories, Andover, Massachusetts). Dialysis was performed with a 1000 Da cut‐off membrane (Spectrum Laboratories, Rancho Dominguez, CA). β‐d‐Galactose penta‐acetate, NAC, EDC, DMAP, *N*,*N*′‐diisopropylethylamine (DIPEA), ethylenediaminetetraacetic acid (EDTA), 5‐hexynoic acid, azido‐PEG4‐amine, copper sulfate pentahydrate, and sodium ascorbate were purchased from Sigma‐Aldrich. Cy5‐mono‐NHS ester was purchased from GE Healthcare (Chicago, IL). All chemicals and solvents were used as purchased without additional purification steps.

#### 3.1.1 Synthesis of dendrimer conjugates

The synthesis of β‐d‐galactose‐PEG4‐azide,^41^ D4‐Gal,^41^ Gal‐D‐Cy5,^41^ and NAC‐NHS ester^58^ was achieved using our recently published protocol.

##### Synthesis of Gal‐D‐NAC

The detailed synthetic procedures for Gal‐D4‐NAC are presented below:

##### Synthesis of d‐hexyne

To a stirring solution of D4‐OH (2 g, 0.14 mmol) in anhydrous DMF (15 ml), hexynoic acid (628 mg, 5.6 mmol) was added. The solution was stirred to dissolve the reactants. It was followed by the addition of DMAP (171 mg, 1.4 mmol) and EDC (1.07 g, 5.6 mmol). The reaction was stirred at room temperature for 12 h. The reaction mixture was then diluted with DMF (50 ml) and dialyzed against DMF for 8 h, followed by the water dialysis for 12 h. The solvents were replaced every 2–3 h. The aqueous solution was then lyophilized to obtain d‐hexyne as white hygroscopic solid in 88% yield.

^1^H NMR (500 MHz, DMSO) δ 8.1–7.7 (m, d‐internal amide *H*), 4.82–4.57 (bs, D‐*OH*), 4.01 (t, ester*‐CH*_2_), 3.44–3.22 (m, D‐*CH*_2_), 3.15–2.85 (m, D and linker‐*CH*_2_), 2.79–2.56 (m, D and linker‐*CH*_2_), 2.47–2.01 (m, D‐*CH*_2_), 1.80–1.57 (m, linker‐*CH*_2_) (Figure S2).

##### Synthesis of D‐hexyne‐NH_2_

To a stirring solution of d‐hexyne (1.6 g, 0.093 mmol) in DMF (5 ml) in a microwave vial, a solution of azido‐PEG4‐amine (392.6 mg, 1.5 mmol) in DMF (2 ml) was added. The solution was stirred for 5 min. This was followed by the addition of a solution of CuSO_4_.5H_2_O (15 mg, 0.06 mmol) in water (1 ml) and sodium ascorbate (12 mg, 0.06 mmol) in water (1 ml). The reaction mixture was stirred in the microwave reactor at 50°C for 8 h. Upon completion, the reaction was diluted with water containing 0.1% EDTA and dialyzed against water to remove the traces of copper. Finally, the dialysis was performed against pure water to remove EDTA. The aqueous solution containing product was lyophilized to obtain d‐hexyne‐NH_2_ in 92% yield.

^1^H NMR (500 MHz, D_2_O) δ 7.85 (s, triazole *H*), 4.57 (t, PEG‐*CH*_2_‐triazole), 4.16 (m, *ester‐CH*_*2*_), 3.94 (t, triazole‐PEG‐*CH*_2_), 3.80–3.53 (m, PEG *H*), 3.47–3.09 (m, D‐*CH*_2_), 2.90–2.34 (m, linker H and D‐*CH*_2_), 2.24 (m, linker‐*CH*_2_), 1.98–1.86 (d m, linker‐*CH*_2_), 1.84–1.72 (m, linker‐*CH*_2_) (Figure S3).

HPLC: Retention time: 13.0 min; Purity: >99% (Figure S4).

##### Synthesis of Gal‐D‐NH_2_

To a stirring solution of d‐hexyne‐NH_2_ (1.7 g, 0.079 mmol) in DMF (5 ml) in a microwave reaction vial, a solution of β‐d‐Galactose‐PEG4‐azide (437.5 mg, 1.106 mmol) in DMF (2 ml) was added. This was followed by the addition of catalytic amount of CuSO_4_.5H_2_O (15 mg, 0.06 mmol) dissolved in water (1 ml) and sodium ascorbate (12 mg, 0.06 mmol) dissolved in water (1 ml). The reaction was stirred in a microwave reactor at 50°C for 8 h. Upon completion, the reaction mixture was diluted with water (100 ml). A solution of ethylenediaminetetracetic acid (EDTA, 1 ml) was added and dialysis was performed against water. The aqueous solution was then lyophilized to obtain the product as white solid in 90% yield.

^1^H NMR (500 MHz, D_2_O) δ 7.88 (s, triazole *H*), 4.59 (t, CH_2_‐triazole), 4.41 (d, *J* = 7.8 Hz, galactose anomeric *H*), 4.20–4.09 (t, CH_2_‐triazole), 3.99–3.52 (galactose *H* and PEG *H*), 3.52–3.17 (m, D‐CH2 and galactose *H*), 3.07–2.34 (D‐*CH*_*2*_ and linker *H*), 1.95 (t, linker *H*) (Figure S5).

HPLC: Retention time: 10.7 min; purity: >99% (Figure S6).

##### Synthesis of Gal‐D‐NAC

To a stirring solution of Gal‐D‐NH2 (1.4 g, 0.05 mmol) in anhydrous DMF (15 ml), DIPEA (1 ml) was added to make the solution slightly basic (pH 7–7.5). A solution of NAC‐NHS ester (364.39 mg, 1 mmol) in DMF (5 ml) was added to the reaction mixture and the stirring was continued for 12 h. The reaction mixture was then diluted with DMF (50 ml) and dialyzed against DMF to remove excess DIPEA and NAC‐NHS. This was followed by the water dialysis to remove DMF. The dialysis solvents were replaced every 2–3 h. The aqueous solution was then lyophilized to obtain Gal‐D‐NAC as white solid in 78% yield.

^1^H NMR (500 MHz, DMSO) δ 8.53–7.69 (m, triazole *H* and D*‐amide H*), 4.71–4.34 (NAC *H*, Gal *H*, and triazole –*CH*_2_), 4.26–3.90 (m, ester‐*CH*_2_, Gal *H*), 3.96–3.78 (m, triazole *H* and *Gal H*), 3.80–3.06 (m, D‐*CH*_2_, PEG *H*, NAC *H*), 3.06–2.62 (m, *D‐CH*_2_ and linker *H*), 2.47–2.10 (m, *D‐CH*_2_ and linker *H*), 1.91 (s, NAC –*CH*_3_) (Figure S7).

HPLC: Retention time: 12.07 min; purity: 98% (Figure S8).

### 3.2 Cell and animal studies

The details about the materials and methods for the cell and animal studies can be found in the supporting information S1.

## 4 CONCLUSIONS

D4‐Gal has significant potential as an intracellular hepatocyte‐specific delivery vehicle in the crowded field of liver‐targeted nanoparticles. There are nanosystems with stronger binding affinities to ASGPR,^69^ higher accumulation in the liver,^70^, ^71^ and a longer demonstrated residence time in the liver^72^; however, no compound has yet been developed that does all three coupled with demonstrated intracellular hepatocyte delivery, not just liver, as we demonstrated specificity through flow cytometry of primary liver cells. From a translational point of view, the present results with D4‐Gal have potential, as the versatility of conjugation chemistries to the hydroxyl surface groups allows for the attachment of small molecules, imaging agents, and potentially small biologics such as siRNA regardless of the payload's charge or aqueous solubility. Additionally, drug conjugates of the vehicle dendrimer used in the present study have shown positive results in Phase 1 and Phase 2a clinical trials. D4‐NAC conjugate (OP‐101) recently completed a Phase 2 trial for hospitalized severe COVID‐19 patients, not only showing improvements in survival compared to standard of care, they successfully treated the hyperinflammation, and significantly reduced blood biomarkers of brain injury in these patients, (NCT04458298),^42^ potentially expediting the clinical translation of Gal‐D‐NAC in the future.

The D4‐Gal conjugate expresses a high affinity for the asialoglycoprotein receptor expressed on liver macrophages, resulting in both increased uptake in HEPG2 cells in vitro and liver tissue in vivo. Localization in the liver is highly specific to hepatocytes, with D4‐Gal present in hepatocytes at a ratio of 25:1 compared to nonparenchymal cells of the liver. Furthermore, there is rapid off‐target clearance of D4‐Gal with no organ except the kidneys containing more than 0.1% of the original injected dose after just 48 h, whereas D4‐Gal was still visible in hepatocytes a week after injection. This preferential liver uptake is maintained in both a mouse model of APAP‐induced liver failure and a rat model of nonalcoholic steatohepatitis. We built upon the impressive hepatocyte‐targeting ability of D4‐Gal when we synthesized Gal‐D‐NAC with an additional 16 molecules of NAC attached to the dendrimer surface via glutathione sensitive linkers for application in a mouse model of severe APAP poisoning. A single intravenous dose of Gal‐D‐NAC improved long‐term survival in APAP mice and showed a significant improvement in liver function and structure by reducing serum aminotransferase levels and restoring hepatocellular organization, even when administered at the delayed time point of 8 h after APAP exposure, while free NAC was ineffective. This indicates that Gal‐D‐NAC may be able to increase the therapeutic window for the thousands of patients that die or require liver transplants due to extreme APAP overdose or delayed treatment.

## AUTHOR CONTRIBUTIONS

**Joshua E. Porterfield:** Conceptualization (equal); data curation (lead); formal analysis (lead); investigation (equal); methodology (equal); project administration (lead); validation (equal); writing – original draft (lead); writing – review and editing (supporting). **Rishi Sharma:** Conceptualization (lead); data curation (lead); formal analysis (equal); investigation (equal); methodology (equal); project administration (equal); supervision (equal); validation (equal); writing – original draft (equal); writing – review and editing (supporting). **Ambar Scarlet Jimenez:** Data curation (supporting); investigation (supporting); methodology (supporting). **Nirnath Sah:** Data curation (supporting); formal analysis (supporting); investigation (supporting). **Sean McCracken:** Investigation (supporting); methodology (supporting). **Lucia Zhang:** Investigation (supporting); methodology (supporting). **Hyoung‐Tae An:** Investigation (supporting); methodology (supporting). **Seulki Lee:** Investigation (supporting); methodology (supporting). **Sujatha Kannan:** Conceptualization (equal); formal analysis (lead); funding acquisition (lead); investigation (lead); resources (lead); supervision (lead); writing – review and editing (equal). **Anjali Sharma:** Conceptualization (equal); data curation (equal); formal analysis (equal); investigation (equal); methodology (equal); writing – original draft (equal); writing – review and editing (supporting). **Kannan Rangaramanujam M.:** Conceptualization (equal); formal analysis (lead); funding acquisition (lead); investigation (lead); resources (lead); supervision (lead); writing – review and editing (lead).

## CONFLICT OF INTEREST

The authors (Rangaramanujam M. Kannan, Sujatha Kannan, Joshua E Porterfield, Rishi Sharma, and Anjali Sharma) have pending patents relating to the hepatocyte targeting and galactose‐dendrimer and PAMAM dendrimer platforms. Rangaramanujam M. Kannan and Sujatha Kannan are co‐founders of Ashvattha Therapeutics Inc., involved with the translation of dendrimer drug delivery platforms. Rangaramanujam M. Kannan and Sujatha Kannan serve as Board of Directors of Ashvattha Therapeutics Inc. till recently, and have financial interests which are managed by Johns Hopkins. Rishi Sharma worked at Ashvattha Therapeutics till recently. This work was done at Johns Hopkins before Rishi Sharma joined Ashvattha Therapeutics.

### PEER REVIEW

The peer review history for this article is available at https://publons.com/publon/10.1002/btm2.10486.

## Supporting information


**Data S1:** Supporting Information

## Data Availability

The data are provided in the supporting information. Additional data are available on request.
