# Supplementary material for: Galactosylated hydroxyl‐polyamidoamine dendrimer targets hepatocytes and improves therapeutic outcomes in a severe model of acetaminophen poisoning‐induced liver failure
Source: Bioeng Transl Med. 2023 Feb 8;8(3):e10486. doi: 10.1002/btm2.10486 (PMC10189448; doi:10.1002/btm2.10486)
Supplement: Supplementary file 1 — Data S1: Supporting Information [file BTM2-8-e10486-s001.docx]

**Supplementary Information**

**Galactosylated hydroxyl-PAMAM dendrimer targets hepatocytes and improves therapeutic outcomes in a severe model of acetaminophen poisoning induced liver failure**

Joshua E Porterfield^1,2#^, Rishi Sharma^1#^, Ambar Scarlet Jimenez^1^, Nirnath Sah^3^, Sean McCracken^1,2^, Lucia Zhang^1,3^, Hyoung-Tae An^1,4^, Seulki Lee^1,4^, Sujatha Kannan^1,3,5^, Anjali Sharma^$^*^1^, and Rangaramanujam M. Kannan*^1,2,5^

^1^Center for Nanomedicine, Department of Ophthalmology, Wilmer Eye Institute Johns Hopkins University School of Medicine, Baltimore, MD 21231, USA;

^2^Department of Chemical and Biomolecular Engineering, Johns Hopkins University, Baltimore MD, 21218, USA;

^3^Department of Anesthesiology and Critical Care Medicine, Johns Hopkins University School of Medicine, Baltimore, MD 21287, USA;

^4^Department of Anesthesiology and Critical Care Medicine, Johns Hopkins University School of Medicine, Baltimore, MD 21287, USA

^5^Hugo W. Moser Research Institute at Kennedy Krieger, Inc., Baltimore MD, 21205, USA

^$^Current address for Anjali Sharma: Department of Chemistry, Washington State University, Pullman, WA 99164, USA.

# These authors contributed equally

*Corresponding authors:

-Rangaramanujam M. Kannan, Arnall Patz Distinguished Professor of Ophthalmology, Center for Nanomedicine at the Wilmer Eye Institute, Johns Hopkins School of Medicine, 400 North Broadway, Baltimore, Maryland 21231, USA;

Tel.: +1 443-287-8634; Fax: +1 443-287-8635; e-mail: [krangar1@jhmi.edu](mailto:krangar1@jhmi.edu)

-Anjali Sharma, Center for Center for Nanomedicine at the Wilmer Eye Institute, Johns Hopkins School of Medicine, 400 North Broadway, Baltimore, Maryland 21231, USA

Tel.: +1 443-469-0033; e-mail: anjali.sharma@wsu.edu

***Materials and methods for cell culture***

***Cell studies.***

Cell Culture Maintenance

HEPG2 cells were purchased from ATCC (Manassas, VA) and utilized between passages 4 and 18. Cells were maintained in a ThermoForma Series II Water Jacketed CO_2_ incubator (ThermoFisher) at 37^o^C and 5% CO_2_ and passaged at 80-90% confluence. Passaging was performed by disengaging the cells with 0.25% trypsin 2.21mM EDTA (Corning, Corning, NY) for 10 minutes at 37^o^C before transfer to a conical tube (Corning) with fresh EMEM medium (ATCC) supplemented with 10% HI-FBS (ThermoFisher). Cells were pelleted at 1,200 rpm for 5 min at 4^o^C in a Sorvall ST 8R centrifuge (ThermoFisher), supernatant was aspirated, and cells resuspended in fresh medium and transferred to a new culture flask (Corning). Medium was changed every 2-3 days during expansion. Cells for toxicity experiments were plated at 2 x 10^5^ cells/well in 96-well plates (Corning), those for uptake experiments were plated at 1.5 x 10^6^ cells/well in 6-well plates (Corning), and imaging studies were performed with cells in 35mm poly-d-lysine coated glass bottom imaging dishes (MatTek, Ashland, MA) seeded with 300 μL of cells at a concentration of 7.5x10^6^ cells/mL. All cells were given 24 hours to adhere to dishes and plates before receiving treatment in cell studies.

Toxicity

HEPG2 cells in triplicates in 96-well plates had medium aspirated and replaced with fresh EMEM medium containing 10% HI-FBS and a 10-fold serial dilution of D4-Gal that had been dissolved and sonicated in EMEM and filtered with a 0.45 µm cellulose acetate centrifuge tube filter (Corning). Cells incubates in D4-Gal containing medium for 24 hours in the incubator before medium was removed and replaced with 100µL of fresh EMEM with no phenol red (HiMedia, West Chester, PA). The MTT (3-(4,5-dimethylthiazol-2-yl)-2,5-diphenyltetrazolium bromide) colorimetric cell viability assay, where the yellow MTT dye is converted to purple formazan in viable cells, was used to assess toxicity. To each well 10µL of MTT (Invitrogen, Carlsbad, CA) dissolved at 5 mg/mL in DPBS (Corning) and sterile filtered was added and pipetted to mix. Cells then incubated an additional four hours at 37^o^C. 85µL was then removed from each well and replaced with 150µL DMSO (Sigma) and thoroughly pipetted to mix. The plates were incubated at 37oC for an additional 10 minutes before each well’s absorbance at λ=540nm was read on a SynergyMx microplate reader (BioTek, Winooski, VT). All reported values are percent viability when compared to healthy, untreated cells from the same plate.

Binding studies

Cell surface receptor binding affinity assays were performed as previously reported [1, 2]. In short, HEPG2 cells at 5 x 106 cells/mL were suspended in ice cold DPBS in 1 mL aliquots in 1.5 mL sample tubes (USA Scientific, Ocala, FL). Tubes were placed on rotator (Thermo) set to 15 rpm at 4^o^C and allowed to equilibrate for 30 minutes. If specific binding was blocked, at this point 3µg of ASGR1 blocking peptide (MyBioSource, San Diego, CA) from 1 mg/mL stock in DPBS was added to each tube and allowed to rotate at 4^o^C for an additional hour. At this point D-Cy5 or Gal-D-Cy5 were added to tubes in a serial dilution providing a range of initial dendrimer concentrations. Tubes were then left to rotate at 4^o^C covered in aluminum foil for six hours. After the six hour incubation, 900 µL from each sample was added to a clean tube and spun down at 1,200 rpm and 4^o^C for 5 minutes on a 5424R benchtop centrifuge (Eppendorf, Hamburg, Germany). Supernatant was aspirated and cell pellets were resuspended in 1mL of ice cold DPBS. This washing process was repeated three times before pellets were resuspended in 200µL of ice cold DPBS and analyzed for fluorescence content on an RF5301PC spectrofluorophotometer (Shimadzu, Kyoto, Japan) at λex=645nm and λem=662nm using a 16.100F-Q-10/Z15 quartz cuvette (Starna Cells, Atascadero, CA). Fluorescence intensities had background cell fluorescence from control samples subtracted off before being converted to dendrimer concentration with previously established calibration curves in DPBS. The bound dendrimer concentration and initial dendrimer concentration for both total and non-specific binding were then converted to KD with a GraphPad 8.3.0 (Prism, La Jolla, CA) program further described elsewhere.[2] Binding studies were performed in triplicate with different passages of HEPG2 cells.

Uptake

6-well plates of HEPG2 cells had their medium removed and replaced with either fresh EMEM or EMEM with 20 mM free β-D-galactose (Sigma) and allowed to incubate for 30 minutes. Then once again medium was removed from each well and replaced with fresh EMEM or EMEM containing 20 μg/mL of D-Cy5 or Gal-D-Cy5 and/or 20mM free galactose. Cells were left to incubate for 24 hours. After sufficient incubation with the dendrimer solutions with or without galactose, medium was removed from all wells and each were washed twice with sterile DPBS at 37^o^C before being released from the plates with trypsin and transferred to separate conical tubes. Each well was then washed with fresh EMEM and added to the same conical tube. The cell count in each well was then taken after dilution in Trypan Blue (Corning) using a hemacytometer (Hausser Scientific, Horsham, PA) on an Eclipse TS100 standing light microscope (Nikon, Tokyo, Japan). Conicals were then spun down at 1,500 rpm and 4^o^C for 5 minutes before supernatant was aspirated and cells were transferred to sample tubes with two 100µL washes of HPLC grade MeOH (VWR, Radnor, PA). Cells in methanol were stored long term at -80^o^C.

Dendrimer extraction was performed by rapid freeze-thaw of cell pellets three times between liquid N2 and a 55^o^C water bath followed by 5 minutes on an 1800 sonicator (Branson Ultrasonics, Danbury, CT). Samples were then spun down at 15,000 rpm for 10 minutes on a benchtop centrifuge, and the supernatants were analyzed for Cy5 fluorescence content on the spectrofluorophotometer at λ_ex_=645nm and λ_em_=662nm. Background fluorescence from untreated cells was subtracted from each sample and then transformed to dendrimer concentration using previously created calibration curves of each batch of D-Cy5 and Gal-D-Cy5 in methanol. Each of the replicates was performed with duplicate treatments that were averaged.

Imaging

HEPG2 cells in imaging wells underwent the same treatments with free galactose and dendrimer as described above. After 24 hours of incubation with dendrimer with or without 20mM free galactose, cells were preserved for fluorescence staining and imaging by first incubating for 10 minutes in 10% formalin neutral buffered solution (Sigma) followed by 5 minutes in Ultrapure H2O (Invitrogen), after which wells received a quick MeOH wash to aid in drying before long term storage at 4^o^C. For staining, cells were first blocked for four hours at room temperature with 1% bovine serum albumin (Sigma) and 0.1% TritonX-100 (Sigma) in 1X TBS (Corning) (TBST) supplemented with 5% normal goat serum (Abcam, Cambridge, UK). Primary stain of 1:250 rabbit α mouse ASGR1 antibody (Proteintech, Rosemont, IL) diluted in TBST was then applied to each imaging dish and incubated overnight at 4^o^C. Wells were washed three times for 5 min each with 1X TBS supplemented with 0.1% TritonX (TBT) before addition of secondary antibody, goat α rabbit 488 (Invitrogen) diluted 1:250 in TBT and incubation for four hours at room temperature. Wells were once again washed thrice with TBT before the addition of NucBlue Live ReadyProbes Reagent Hoechst 33342 (Invitrogen) for 10 minutes. Wells were finally washed twice in TBT and once in TBS before long term storage at 4^o^C. Imaging was performed on an LSM 710 confocal microscope running ZEN software (Zeiss, Oberkochen, Germany) using laser levels set with an untreated cell imaging dish. Imaging studies were performed in triplicate to ensure replicability of published images.

Statistics

All data collection was performed in a blinded manner, and analysis was performed in GraphPad 8.3.0 software (Prism). Toxicity studies were analyzed by one-way ANOVA with Geisser-Greenhouse correction of unequal variance due to the varying sample size per treatment group. Dendrimer uptake data analysis utilized a two-way ANOVA to determine a main effect of the interaction of dendrimer and blocking flowed by Tukey’s multiple comparison t-tests. Binding was determined with the “One site -- Total and non-specific binding” nonlinear regression analysis on GraphPad with background set to zero. In this study p<0.05 is represented by *, p<0.01 by **, p<0.001 by ***, and p>0.05 by n.s.

***Animal studies for biodistribution and treatment.***

Animals

All protocols performed in this study were approved by the Johns Hopkins University Animal Care and Use Committee (protocols MO18M128 and RA18M197). Male and female C57BL6 mice were purchased from Jackson Laboratory (Bar Harbor, ME) at 5-6 weeks of age and used between 6 and 8 weeks of age. Male and female 6-7 week old Sprague-Dawley rats were purchased from Charles River (Wilmington, MA) and used at 12-13 weeks of age. Experiments were performed with a roughly equal number of male and female animals. All animals were housed in central facilities at 22°C, 50% relative humidity and 12-h light/dark cycle.

Injections

Injections of dendrimer were prepared at a concentration of 11 mg/mL in 0.9% sodium chloride injection, USP (Baxter, Deerfield, IL) and sterilized through Spin-X centrifuge tube filters (Corning). Animals received 55 mg/kg dose of dendrimer (~100 µL injection volume) via insulin syringe (SureComfort, South Korea) into the tail vein under 2% isoflurane (Baxter) provided by a vaporizer (Kent Scientific, Torrington, CT). Prior to and following injection, tails were sterilized with alcohol swabs (Becton Dickinson, Franklin Lakes, NJ). Animals were kept warm with a heat lamp throughout the procedure and as they recovered from anesthesia. Any animals expressing adverse reactions to the injection (of which there were none) would have been immediately removed from the study.

Biodistribution

Animals receiving a 55 mg/kg dose of either D-Cy5 or Gal-D-Cy5 were sacrificed by anesthesia overdose followed by cardiac puncture either 1, 4, 24, or 48 hours after dendrimer administration. Whole blood was drawn from the heart with a 1mL TB syringe with a 26G needle (Becton Dickinson) and placed in a 1.5 mL Hep-Li coated heparinized tube (Milian, Vernier, Switzerland), shaken, and spun down on a benchtop centrifuge to separate plasma and cells. Animals were then perfused with 10-30mL of DPBS through the right atrium and flowing out through the snipped visceral vena cava. Perfusion was considered complete when the liver completely dulled in color and the perfusate ran clear. Internal organs were then dissected out whole and flash frozen if destined for dendrimer extraction or placed in formalin (4% paraformaldehyde) overnight if meant for use in sectioning and imaging. Preservation in formalin was followed by 24 hours each in 10, 20, and 30% sucrose (Sigma) solution in DPBS with 0.1% NaN3 (Sigma) before being embedded in OCT (Sakura Finetek, Torrance, CA) and stored at -80^o^C. Livers were preserved for imaging with a four hour incubation in 2% PFA (Electron Microscope Sciences, Hatfield, PA), 0.1M lysine (BulkSupplements, Henderson, NV) 0.02 mg/mL NaIO4 (Sigma) in DPBS solution for four hours, after which samples were washed with 0.5M sucrose in DPBS twice for 10 minutes each and stored in 0.5M sucrose at 4oC overnight. Finally, liver samples were put into 1:1 OCT:0.5M sucrose for an additional 24 hours at 4oC before being embedded in OCT and stored at -80^o^C.

Quantification of dendrimer uptake in tissues was performed by massing the whole organ on an XPE105 balance (Mettler Toledo, Columbus, OH) prior to isolating individual samples of each organ for homogenization (three 100±5mg samples of brain, two 40±5mg samples of heart, three 60±5mg samples of kidneys, three 100±5mg samples of liver, three 40±5mg samples of lungs, and the spleen as a single whole sample). Tissue samples then had a scoop of 0.9-1.4 mixed stainless steel homogenization beads (NextAdvance, Troy, NY) and 1 mL of MeOH per 100 mg of tissue added to each sample vial and sealed. Samples were placed on a Storm 24 Bullet Blender (NextAdvance) and homogenized for 10 minutes at power level 6 for soft tissues and level 12 for tougher tissues. Homogenized samples were then spun down at 15,000 rpm for 10 minutes and the supernatant transferred to a 2mL protein lo-bind tube (Eppendorf) for long term storage at -80^o^C. Fluorescence quantification of samples was performed on a spectrofluorophotometer set to λ_ex_=645nm and λ_em_=662nm and net fluorescence intensities were calculated by subtracting fluorescence from tissue from non-injected animals. Net intensities were converted to dendrimer concentration and %ID via predetermined calibration curves for each batch of dendrimer in MeOH. Each replicate in the data presented is the average of all the samples of an organ from a single animal.

IVIS® optical imaging:

Flash frozen whole livers were allowed to thaw on ice before being placed on disposable petri dishes (ThermoFisher). Fluorescence imaging was performed on an IVIS Spectrum optical imaging device (Xenogen, Alameda, CA) on the same day with the same settings. Image analysis was performed on Living Image software (Caliper Life Sciences, Waltham, MA)

Immunohistochemistry and confocal imaging

Preserved organs frozen in OCT were sectioned on a CM3050S cryostat (Leica, Wetzlar, Germany) with livers cut at 6 µm and all other organs cut at 20 µm. Organ sections were stored at -20^o^C prior to immunohistochemical staining at which point slides were allowed to come to room temperature prior to a 5-minute rehydration in TBS. Liver sections were blocked with 1X TBS containing 5% BSA, 0.1% saponin (Sigma), and 0.1% Triton X for four hours at room temperature. All other tissues were blocked with TBST supplemented with 5% NGS for four hours at room temperature. Blocking buffer was then aspirated and primary antibody was applied diluted in blocking buffer overnight at 4oC. Primary antibodies utilized in this study and their dilutions are as follows: rabbit α mouse serum albumin (1:50, Abcam), rabbit α mouse ASGR1 (1:250, Proteintech), mouse α human sinusoidal endotheial cell 1 (SE-1, 1:500, Novus, St. Charles, MO), and Isolectin GS-IB4 Alexa Flour 488 (Invitrogen). The following day, slides were washed three times for 5 minutes each in TBT before secondary antibody was applied diluted in TBT for four hours at room temperature. Secondary antibodies utilized in this study were: goat α rabbit 488 (1:250, Invitrogen), goat α rabbit Cy3 (1:250, Invitrogen), goat α mouse 488 (1:250, Invitrogen), and goat α mouse Cy3 (1:250, Invitrogen). Slides were then washed three times for 5 minutes each with TBT before application of Hoechst 33342 for 10 minutes. A final two washes in TBT and one wash in TBS was performed before slides were mounted with Dako fluorescence mounting medium (Agilent, Santa Clara, CA) and stored at 4^o^C. Imaging was performed on a Zeiss LSM 710 confocal microscope and all laser and picture settings were based on unstained/uninjected background tissue prepared in the same manner.

Flow cytometry

Protocol for isolation of primary liver cells was followed as published previously.[2] Fresh perfusion solutions were prepared as follows. Solution 1: 0.5mM EDTA (ThermoFisher) in Hank’s Balanced Salt Solution (Gibco, Gaithersburg, MD). Solution 2: DMEM (Gibco) supplemented with 5% HI-FBS and 0.8 mg/mL collagenase type I (Worthington, Lakewood, NJ). Solution 3: DMEM with 5% HI-FBS. Mice injected with 55 mg/kg D-Cy5 or Gal-D-Cy5 24 hours prior were deeply anesthetized with 5mL/kg 25% ketamine (Vedco, St. Joseph, MO) 2.5% xylazine (MWI Veterinary Supply, Boise, ID) in 4:1 saline:EtOH (Pharmco, Toronto, Canada) until non-responsive to toe pinch. Mice were then placed on a bench under a heat lamp, had the chest cavity opened, and a 25G needle connected to the outlet of a 205S perfusion pump (Watson Marlow, Seaford, DE) connected to Solution 1 in a hot water bath inserted into the right atrium. Perfusion was then started at 5 mL/min with the visceral vena cava severed once the liver began to visibly inflate. Perfusion with Solution 1 for 7 minutes was followed by perfusion with warm Solution 2 for 7 minutes after which the liver was dissected out and placed in 15mL of warm Solution 2. The liver in Solution 2 was then poured into a 100 µm nylon mesh cell strainer (ThermoFisher) on a 50 mL conical and pulverized with the plunger of a 10 mL syringe as fresh Solution 3 was poured over. The cell suspension was then spun down at 50 rpm for 1 minute and the supernatant carefully aspirated. The cells were washed once more in Solution 3 before being resuspended in 20 mL of Solution 3 and counted using a Countess II cell counter (Life Technologies, Carlsbad, CA). Cells were spun down and resuspended at 1 x 10^6^ cells/mL in ice cold flow cytometry staining (FACS) buffer (Invitrogen).

100 µL of cell suspension was added in duplicate to wells of a 96-well round bottom plate (Corning) and spun down at 1,500 rpm and 4^o^C for 5 minutes on a Sorvall Legend X1R centrifuge (ThermoFisher). All cells not receiving stains were still washed and resuspended in buffers not containing stains in the following steps. Cells were washed twice more with ice cold FACS buffer before being resuspeded in 100 µL of ice cold DPBS with eBioscience fixable viability dye eFluor 450 (ThermoFisher) diluted at 1:1000 to incubate at 4^o^C for 30 minutes. Then cells were washed twice with DPBS and resuspended in 20 µL of human TruStain FcX (BioLegend, San Diego, CA) diluted 1:50 in FACS buffer. After 10 minutes at room temperature 20 µL of rabbit α mouse ASGR antibody (Proteintech) diluted 1:50 in FACS buffer was added to each well and allowed to incubate for an additional 20 minutes at room temperature. Cells were then washed twice in FACS buffer and resuspended in 40 µL of goat α rabbit 488 (Invitrogen) diluted 1:250 in FACS buffer for 30 min at room temperature. Two final washes with 100 µL of FACS buffer occurred before each sample was transferred to its own 5 mL round bottom tube with 1 mL of FACS buffer. Cells suspensions were then analyzed on an SH800S Cell Sorter (Sony, San Jose, CA). Analysis was performed in FlowJo 10.6.1 (Becton Dickinson). The population of individual cells was gated based on FSC v. SSC before live cells (450 negative), hepatocytes (488 positive), non-hepatocytes (488 negative), and dendrimer-containing cells (Cy5 positive) were sequentially identified based on gating from unstained/uninjected tissues.

Model of severe acetaminophen poisoning induced liver failure

Model development was based on previous studies to titrate a dose of acetaminophen (APAP, Sigma) to achieve high rates of mortality within 96 hours [2-4]. Animals were massed and began food restriction 16 hours before poisoning, but were given water ad libitum. APAP was prepared fresh in a 10% DMSO 90% 0.9% sodium chloride injection, USP solution at a concentration of 25 mg/mL. Mice were massed just prior to injection and received 800 mg/kg APAP intraperitoneally via insulin syringe (Becton Dickinson). Insulin syringe was held in the intraperitoneal cavity for 30 seconds to ensure no backflow of drug and animals were returned to the cage and monitored. Two hours after APAP administration, food was returned and regular mass monitoring began. Animals were removed from the study and sacrificed when their mass dropped below 80% of their initial pre-starvation mass. Dendrimer, free NAC (Sigma), and saline treatments were administered via tail vein injection 8 hours following APAP overdose. Animal sacrifice and tissue collection was performed as above, but with plasma samples flash frozen, and a fresh liver sample homogenized in complete EDTA-free protease inhibitor (Roche, Mannheim, Germany) diluted 1:25 in Tissue Protein Extraction Reagent (Thermo) prior to flash freezing.

Non-alcoholic steatohepatitis

Sprague-Dawley rats were given a methionine-choline deficient, high-fat diet (A06071302, Research Diets, New Brunswick, NJ) for 6 weeks to induce non-alcoholic steatohepatitis. Rats were then administered 20 mg/kg of D-Cy5 or Gal-D-Cy5 via tail vein and sacrificed 24 hours later. Animals were perfused and liver tissue was isolated and flash frozen for later homogenization and extraction of dendrimer.

Hematoxylin & Eosin staining of liver sections

Liver samples were fixed, sectioned and stained with hematoxylin-eosin according to standard procedures. The stained slides were imaged at 10X for histological quantification of nuclear pyknosis, vacuolation and cytoplasmic hypereosinophilia. For scoring these histological changes, we used graded evaluation method ranging from 0-4 signifying level of injury based on the extent of nuclear pyknosis, vacuolation and cytoplasmic hypereosinophilia.[5] Individual scores from each of these three histological changes, as indicated below (Table 1), were summed up to provide a composite liver injury score. 6-7 sections from 2 untreated and 2 treated mice were compared.

Table 1: Semi quantitative liver injury scoring:

| **Injury assessment score** | **0** | **1** | **2** | **3** | **4** |
| --- | --- | --- | --- | --- | --- |
| Nuclear pyknosis | None | ≤5% of all cells | 6-15% of all cells | 16-30% of all cells | >30% of all cells |
| Cytoplasmatic vacuolation | None | <20% of all nucleated cells | 20-50% of all nucleated cells | 51-80% of all nucleated cells | >80% of all nucleated cells |
| Cytoplasmatic hypereosinophilia | None | ≤5% of field of view | 6-10% of field of view | 11-20% of field of view | >20% of field of view |

HNE staining

Saline perfused liver tissue was saved in 10% formalin overnight, then paraffin sectioned. Liver tissue sections were deparaffinized, washed in PBS, and incubated in sodium citrate solution for 20 min at 100 °C for antigen retrieval. After another PBS wash, the sections were treated with 3% H_2_O_2_, followed by a blocking buffer (5% goat serum, 0.5 % Triton X-100 in 1x PBS) for two hours at room temperature. Sections were then incubated overnight at 4 °C with a rabbit anti-4HNE primary antibody (1:600; Alpha Diagnostics International, TX, USA). Following primary antibody incubation, sections were washed thoroughly with PBS and incubated with HRP-conjugated goat-anti-rabbit antibody in the blocking buffer. Subsequently, streptavidin horseradish peroxidase and then with 3,3-diaminobenzidine HRP substrate were added to the sections (Vector Laboratories, USA) for up to five minutes. Stained sections were appropriately mounted onto microscope slides for imaging.

The 4-HNE images were acquired at 20X magnification. All the image analyses were done in image j. The images were thresholded to detect 4-HNE positive granules and particle analysis function was used to quantify their number. Liver sections from 3 healthy control, 2 untreated, and 2 treated mice were stained for 4-HNE and analyzed.

Biochemical analysis

Plasma samples were allowed to thaw on ice before alanine aminotransferase (ALT) and aspartate aminotransferase (AST) were analyzed with colorimetric aminotransferase activity kits (Sigma). All samples were run in duplicate. In brief, for ALT, 3 µL of serum from each animal was placed in the well of a 96-well plate and brought to a final volume of 20µL with assay buffer, followed by 100 µL of master reaction mixture. The plate was then incubated at 37^o^C for three minutes before a microplate reader was used to read the absorbance in each well at λ=570nm every 5 minutes until the samples exceeded the standards after which the standards were used to convert absorbance change to ALT activity. The AST assay was performed similarly with the appropriate assay buffer and master reaction mixture. 8 µL of serum from each animal was added to wells of a 96-well plate and brought to a final volume of 50 µL with assay buffer. 100 µL of master reaction mixture was added to each well and mixed. The plate was incubated at 37^o^C for three minutes before a microplate reader was used to read the absorbance in each well at λ=450nm every 5 minutes until the samples exceeded the standards after which the standards were used to convert absorbance change to AST activity.

**Figure S1.** Biochemical effect of Gal-D-NAC treatment on clinical liver function markers. **A**. Serum alanine aminotransferase; **B**. aspartate aminotransferase levels in both healthy mice and those receiving acetaminophen overdose and treatment with saline, free N-acetyl cysteine, or Gal-D-NAC. No significant difference in ALT and AST was seen between the groups (for serum ALT, p= 0.0871 for untreated vs healthy; p=0.514 for healthy vs Gal-D-NAC and p=0.9918 for free NAC vs untreated groups).

Statistics

Statistical analyses were done in Prism 8.3.0 software (GraphPad). Uptake comparisons between dendrimers in tissues as well as flow cytometry data were performed with two-way ANOVAs followed by Tukey’s comparative t-tests. Uptake in disease models was also first analyzed with a two-way ANOVA followed by comparison within diseases using Sidaks’ multiple comparison t-test. Biochemical markers were analyzed with one-way ANOVAs followed by Tukey’s comparative t-tests. Equal numbers of each gender were used when possible, and images are representative of at least three biological replicates unless otherwise stated. p=0.05 was used in all a priori tests and p=0.0167 as the Bonferroni-corrected value to correct the alpha level when multiple comparisons were performed. In figures, means are plotted with error bars representing the standard error of the mean, and * represents p < 0.05, ** p<0.01, *** p<0.001, and n.s. p > 0.05.

***Characterization of intermediates and dendrimer conjugates.***

**Figure S2.** ^1^H NMR of D-hexyne (DMSO-*d6*).

**Figure S3.** ^1^H NMR of D-hexyne-NH_2_ (D_2_O).


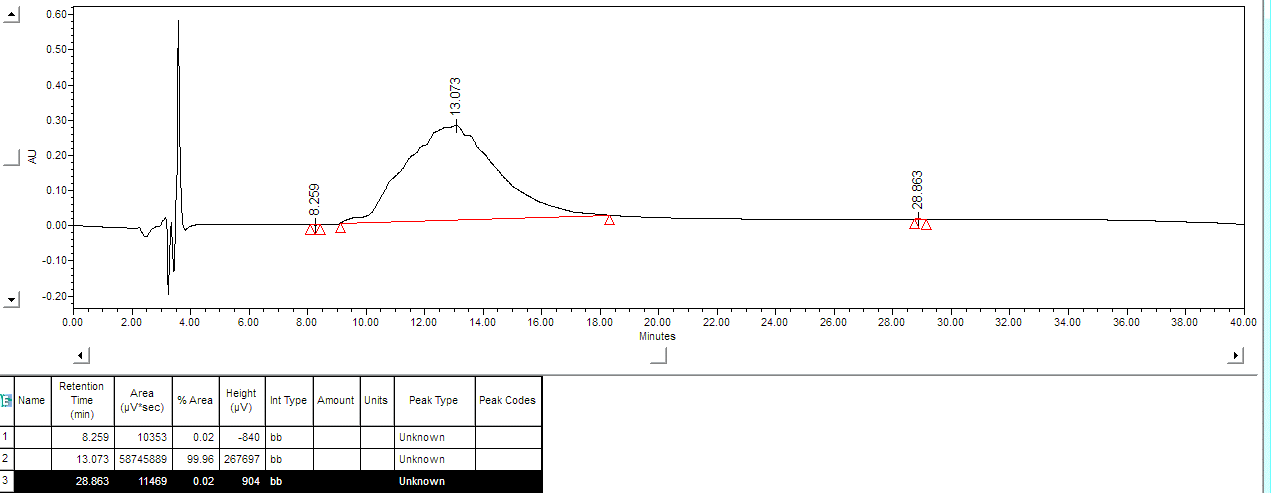


**Figure S4.** HPLC chromatogram of D-hexyne-NH_2_.

**Figure S5.** ^1^H NMR of Gal-D-NH_2_ (D_2_O).


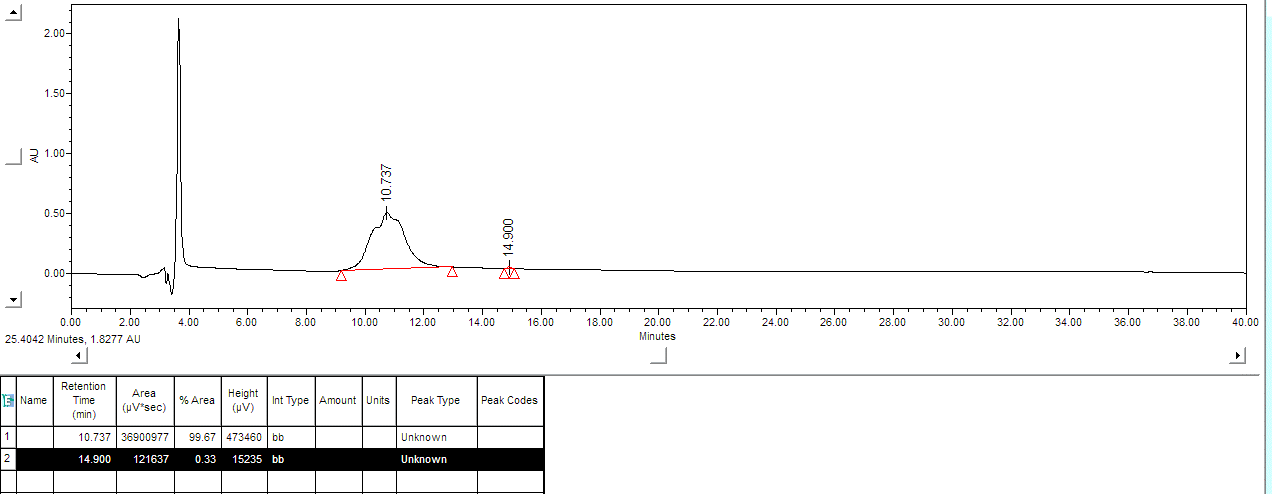


**Figure S6.** HPLC chromatogram of Gal-D-NH_2_.

**Figure S7.** ^1^H NMR of Gal-D-NAC (DMSO-*d6*).


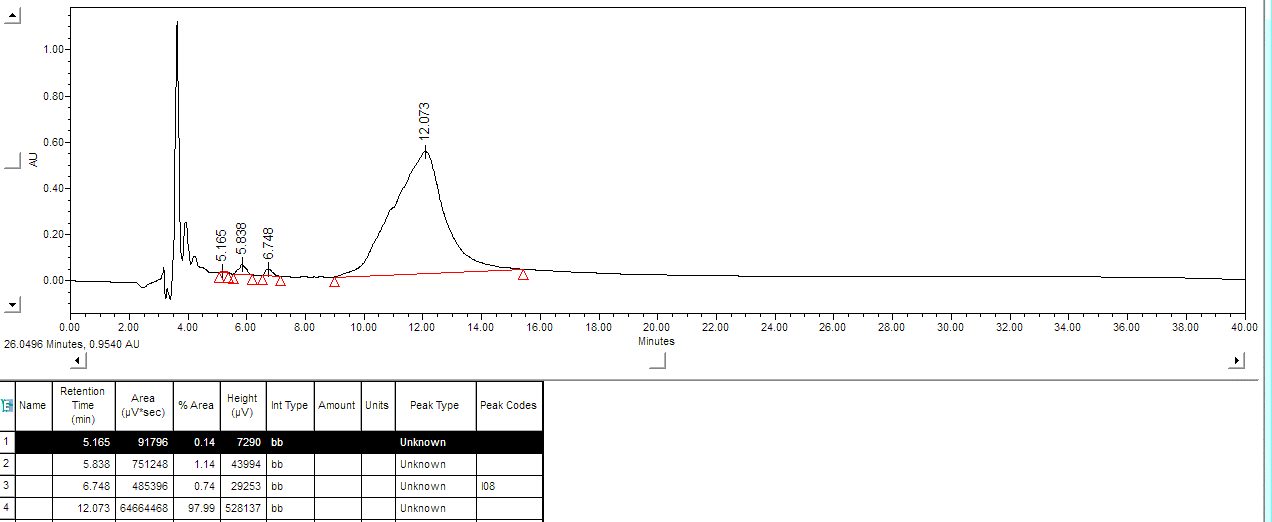


**Figure S8.** HPLC chromatogram of Gal-D-NAC.


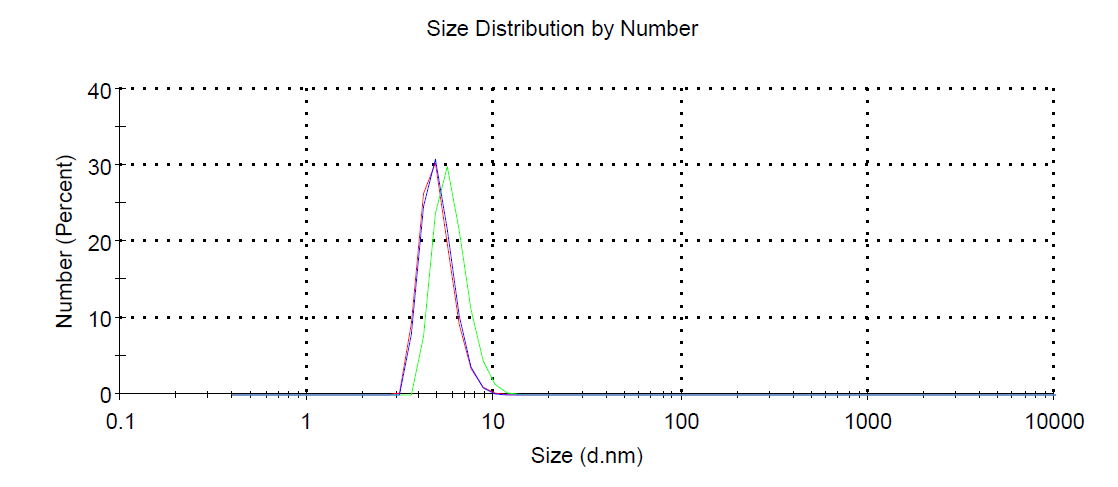


**Figure S9**. Size distribution of Gal-D-NAC determined by dynamic light scattering (5.3 nm)


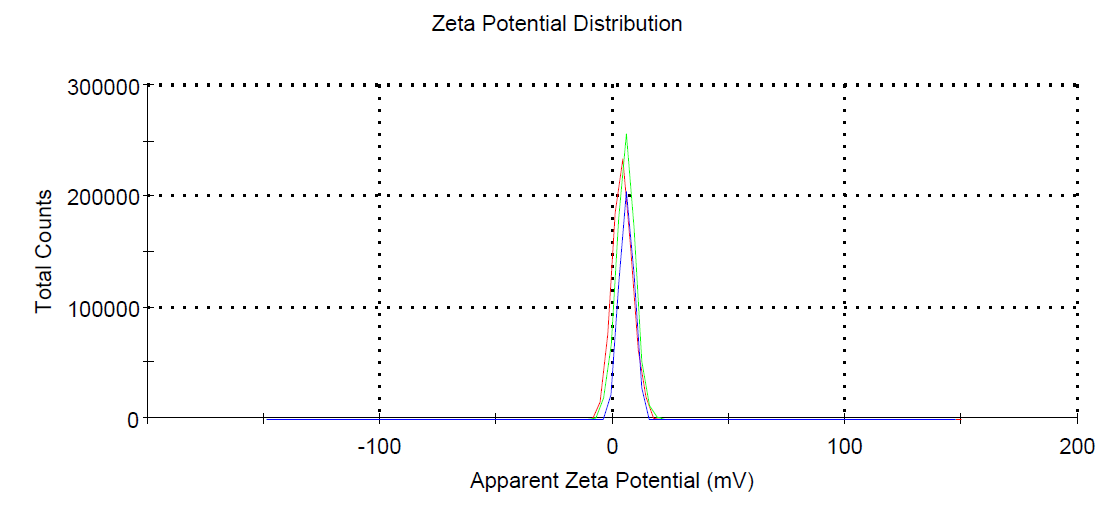


**Figure S10**. Zeta potential distribution of Gal-D-NAC (+5.1 mV)

References

[1] M. Sempkowski, C. Zhu, M.Z. Menzenski, I.G. Kevrekidis, F. Bruchertseifer, A. Morgenstern, S. Sofou, Sticky Patches on Lipid Nanoparticles Enable the Selective Targeting and Killing of Untargetable Cancer Cells, Langmuir 32(33) (2016) 8329-8338.

[2] R. Sharma, J.E. Porterfield, H.-T. An, A.S. Jimenez, S. Lee, S. Kannan, A. Sharma, R.M. Kannan, Rationally Designed Galactose Dendrimer for Hepatocyte-Specific Targeting and Intracellular Drug Delivery for the Treatment of Liver Disorders, Biomacromolecules 22(8) (2021) 3574-3589.

[3] H. Jaeschke, Y. Xie, M.R. McGill, Acetaminophen-induced Liver Injury: from Animal Models to Humans, J Clin Transl Hepatol 2(3) (2014) 153-161.

[4] J. Ward, S. Bala, J. Petrasek, G. Szabo, Plasma microRNA profiles distinguish lethal injury in acetaminophen toxicity: a research study, World J Gastroenterol 18(22) (2012) 2798-2804.

[5] N. Tapuria, S.P. Junnarkar, N. Dutt, M. Abu-Amara, B. Fuller, A.M. Seifalian, B.R. Davidson, Effect of remote ischemic preconditioning on hepatic microcirculation and function in a rat model of hepatic ischemia reperfusion injury, HPB 11(2) (2009) 108-117.
